# The global prevalence of fusidic acid resistance in clinical isolates of *Staphylococcus aureus*: a systematic review and meta-analysis

**DOI:** 10.1186/s13756-021-00943-6

**Published:** 2021-05-01

**Authors:** Bahareh Hajikhani, Mehdi Goudarzi, Sareh Kakavandi, Sana Amini, Samira Zamani, Alex van Belkum, Hossein Goudarzi, Masoud Dadashi

**Affiliations:** 1 Department of Microbiology, School of Medicine, Shahid Beheshti University of Medical Sciences, Tehran, Iran; 2 Data Analytics Unit, bioMérieux 3, Route de Port Michaud, La Balme Les Grottes, France; 3 Department of Microbiology, School of Medicine, Alborz University of Medical Sciences, Karaj, Iran; 4 Non-Communicable Diseases Research Center, Alborz University of Medical Sciences, Karaj, Iran

**Keywords:** Fusidic acid, *Staphylococcus aureus*, MRSA, Meta-analysis

## Abstract

**Background and aim:**

*Staphylococcus aureus* (*S. aureus*) is one of the most common pathogens causing nosocomial and community-acquired infections with high morbidity and mortality rates. Fusidic acid has been increasingly used for the treatment of infections due to methicillin-susceptible *S. aureus* (MSSA) and methicillin-resistant *S. aureus* (MRSA). The present study aimed to determine the precise prevalence of fusidic acid resistant MRSA (FRMRSA), fusidic acid resistant MSSA (FRMSSA), and total fusidic acid resistant *S. aureus* (FRSA) on a global scale.

**Methods:**

Several international databases including Medline, Embase, and the Web of Sciences were searched (2000–2020) to discern studies addressing the prevalence of FRSA, FRMRSA, and FRMSSA. STATA (version14) software was used to interpret the data.

**Results:**

Of the 1446 records identified from the databases, 215 studies fulfilled the eligibility criteria for the detection of FRSA (208 studies), FRMRSA (143 studies), and FRMSSA (71 studies). The analyses manifested that the global prevalence of FRSA, FRMRSA, and FRMSSA was 0.5%, 2.6% and 6.7%, respectively.

**Conclusion:**

This meta-analysis describes an increasing incidence of FRSA, FRMSSA, and FRMRSA. These results indicate the need for prudent prescription of fusidic acid to stop or diminish the incidence of fusidic acid resistance as well as the development of strategies for monitoring the efficacy of fusidic acid use.

**Supplementary Information:**

The online version contains supplementary material available at 10.1186/s13756-021-00943-6.

## Introduction

*Staphylococcus aureus* (*S. aureus*) is one of the most important causative agents in a wide range of clinical syndromes, from the skin and soft tissue infections to infective endocarditis and bacteremia as well as different prosthetic device-related infections which are reported largely worldwide [1–3]. This bacterial species can become resistant to many antibiotics, using different mechanisms. Acquisition of mobile genetic resistance elements by horizontal gene transfer, mutations of antibiotic targets, and overexpression of endogenous efflux pumps are among the most important mechanisms [4–6]. Knowing how antibiotics work and how bacteria become resistant, as well as generating accurate statistics on the rate of antibiotic resistance in different parts of the world, are important for medical management and therapeutic decisions. Furthermore, it can help in the development of practical infection control methods as well as the prevention of bacterial resistance spreading. Fusidic acid (FA) is one of the important antibacterial agents that requires ongoingevaluation of its mode of action and assessment of its resistance rate to help improve treatment strategies, particularly in the caseof staphylococcal infections. FA, derived from the fungus *Fusidium coccineum* (in 1960), shows moderate activity against most Gram-positive bacteria including staphylococci and also covering methicillin-resistant *S. aureus* (MRSA), and some anaerobic Gram-negative organisms [7]. It is generally used topically for the treatment of *S. aureus* skin infections. Different creams and ointments containing FA are commercially available. Intravenous and oral preparations of this antibiotic are also used as anti-staphylococcal agents to treat persistent skin infections as well as chronic bone and joint infections [7, 8]. In addition, FA is an important and valuable alternative to vancomycin to combat resistant organisms [4]. FA primarily has bacteriostatic effects, although it may be bactericidal in high concentrations [9]. FA is a specific inhibitor of the elongation factor G (EF-G) which is essential in the peptide translocation step during protein synthesis. FA inhibits the GTPase function of EF-G after binding to its target and then prevents further elongation of the polypeptide chain [10]. Plasmid mediated resistance resulted from decreased bacterial cell wall or membrane permeability. Chromosomal mutations expressed in EF-G and its associated proteins (fusB, fusC, and fusD) as well as a mutation in *fusA,* and *fusE* genes have been described as relevant factors in FA resistance [11, 13] The lack of cross-resistance with other important classes of antibiotics (beta-lactams, macrolides and, aminoglycosides) is an important feature of this antibiotic which may be due to the widely different chemical structures of these agents [10]. Nonetheless, increased antibiotic usage, combined with increased therapy duration, has been linked to an increased rate of FA resistance among *S. aureus* isolates [12]. FA resistance rates were evaluated in 13 European countries. The results of this survey showed that the overall prevalence of resistance to FA was 10.7% among *S. aureus* isolates. The highest rate of resistance (62.4%) was observed among isolates from Greece [14]. Although several studies have examined the resistance FA among of *S. aureus* strains, a comprehensive study reporting global data is not available. So, the current study aims to evaluate the dissemination and prevalence of all FA resistant *S. aureus* (FRSA), and also FA resistant MRSA (FRMRSA), and FA resistant MSSA (FRMSSA), among clinical isolates in a meta-analysis and systematic review.

## Methods

### Literature search

A systematic search was conducted to evaluate the prevalence of FRSA among clinical strains based on selected keywords (*Staphylococcus aureus*,* Staphylococcus*, *S. aureus,* fusidic acid, sodium fusidate, and fucidin) using three main electronic databases including Medline (via PubMed), Embase, and Web of Science (2000–2020). Original articles published in English that provided the prevalence or incidence of FRSA, FRMRSA, and FRMSSA were selected for further analysis. We also searched the bibliographies for additional relevant articles.

### Inclusion and exclusion criteria

All original papers presenting cross-sectional studies on the prevalence of FRSA, FRMRSA, and FRMSSA were included. All selected studies were screened based on titles, abstracts, and full texts consecutively. Studies were included in our analysis based on the following criteria: (1) original articles that provided sufficient data on FRSA; (2) used standard methods; (A) disk diffusion method; (B) agar dilution, microdilution, and macrodilution methods or E-test; and (C) molecular methods to detect FRSA, FRMRSA and FRMSSA according to the CLSI (2020) guidelines [15]. The exclusion criteria were: (1) articles studying non-human samples; (2) studies considering; (A) FA resistant bacteria except *S. aureus*; (B) other types of antibiotic resistance except FA; (3) review articles; (4) abstracts reported in conferences; and (5) duplicate article.

### Data extraction and definitions

The author’s last name, date(s) of the investigation, year of publication, country/continent, total number of *S. aureus*, MRSA, MSSA, FRSA, FRMRSA and FRMSSA as well as a detection method and the source of isolates were extracted from the enrolled studies. The prevalence of FRSA, FRMRSA, and FRMSSA isolates was evaluated as well. Two independent researchers recorded the data to avoid bias.

### Quality assessment

All evaluated studies were subjected to a quality assessment (designed by the Joanna Briggs Institute), and only high-quality ones were selected for our final analysis [16].

### Meta-analysis

STATA (version 14.0) software was used to analyze the extracted data. The data were pooled using the fixed-effects model (FEM) [17] and the random-effects model (REM) [18]. Statistical heterogeneity was assessed using the Cochran Q and I2 statistical methods [19].

## Results

### Characteristics of included studies

**Fig. 1:**
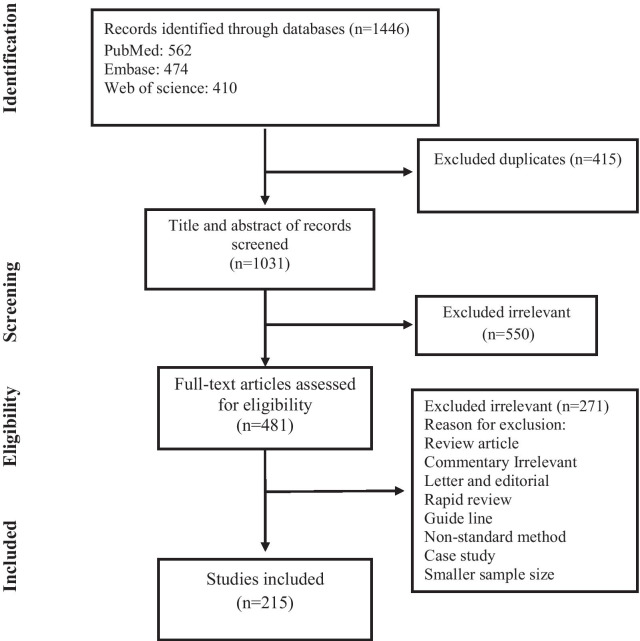
Flow chart of study selection for inclusion in the systematic review and meta-analysis

After removing duplicates, we identified a total of 1446 articles in the databases. Based on the title and abstract evaluation in secondary screening, 471 of the chosen ones were excluded (see Fig. 1 which also includes the reasons for rejection). In the next step, upon full text research, 208, 143, 71 articles were included for FRSA, FRMRSA, and FRMSSA, respectively [11, 20–233]. The characteristics of the included articles are shown in Additional file 1: Tables S1, S2, and S3.

### The prevalence of FRSA, FRMRSA and FRMSSA among clinical isolates

The pooled and averaged prevalence of FRSA, FRMRSA and FRMSSA were 0.5 [(95% CI) 4.6–5.4] among 157,220 *S. aureus* isolates, 2.6 [(95% CI) 2.3–2.9] among 94,238 *S. aureus* isolates, 6.7 [(95% CI) 5.4–7.9] among 11,992 *S. aureus* isolates, respectively. Also, the pooled prevalence of FRMRSA among 50,078 MRSA isolates and FRMSSA among 70,438 MSSA isolates was 5.8 [(95% CI) 5.0–6.6] and 3.0 [(95% CI) 2.6–3.5], respectively (Tables 1, 2).

**Table 1:** Prevalence of *FRSA, FRMRSA* and *FRSSA* based on study periods and continents

Category	Subcategory	No. studies	No. strains	Prevalence (%) (95% CI)
*FRSA*				
Overall	*FRSA*/*S. aureus*	120	7562/157,220	0.5 (4.6–5.4)
Study period	Before 2000	26	3478/90,563	4.0 (3.4–4.5)
	2000–2009	55	2834/46,134	5.6 (4.9–6.2)
	2010–2020	39	1250/20,523	5.2 (4.4–6.1)
Continent	Asia	32	768/11,239	5.6 (4.6–6.6)
	Europe	69	5519/122,267	4.7 (4.3–5.2)
	America	9	408/7010	5.5 (3.4–7.5)
	Africa	3	58/723	5.5 (0.6–10.4)
	Oceania	7	809/15,981	5.0 (3.7–6.3)
*FRMRSA*				
Overall	*FRMRSA*/*S. aureus*	79	2193/94,238	2.6 (2.3–2.9)
Study period	Before 2000	14	547/39,499	1.4 (1.1–1.8)
	2000–2009	35	1094/41,157	2.9 (2.3–3.4)
	2010–2020	30	552/13,582	3.2(2.3–4.1)
Continent	Asia	27	296/7330	3.0 (2.1–4.0)
	Europe	36	1449/77,695	1.9 (1.5–2.2)
	America	7	302/4798	4.3 (1.5–7.2)
	Africa	6	111/1334	6.8 (3.6–9.9)
	Oceania	3	35/3081	1.1 (0.8–1.5)
Overall	*FRMRSA*/*MRSA*	72	3211/50,078	5.8 (5.0–6.6)
Study period	Before 2000	19	1410/20,768	5.9 (4.2–7.5)
	2000–2009	31	1127/20,601	5.0 (4.0–6.1)
	2010–2020	22	674/8709	6.8 (5.3–8.3)
Continent	Asia	26	1155/11,414	6.4 (4.4–8.3)
	Europe	33	1637/32,906	1.9 (1.5–2.2)
	America	7	295/4130	5.3 (3.2–7.4)
	Africa	2	5/138	3.6 (0.5–6.7)
	Oceania	4	119/1490	1.1 (0.8–1.5)
*FRMSSA*				
Overall	*FRMSSA*/*S. aureus*	9	939/11,992	6.7 (5.4–7.9)
Study period	Before 2000	2	432/5425	8.0 (7.2–8.7)
	2000–2009	4	489/6153	7.1 (5.3–8.9)
	2010–2020	3	18/414	8.0 (7.2–8.7)
Continent	Asia	2	9/227	4.0 (1.1–7.0)
	Europe	6	923/11,550	7.8 (6.8–8.7)
	America	NR	NR	NR
	Africa	NR	NR	NR
	Oceania	1	7/215	3.3 (9.0–5.6)
Overall	*FRMSSA*/*MSSA*	42	2437/70,438	3.0 (2.6–3.5)
Study period	Before 2000	24	1825/57,798	2.9 (2.5–3.4)
	2000–2009	10	497/8051	3.8 (1.6–6.1)
	2010–2020	8	115/4589	2.8 (1.8–3.7)
Continent	Asia	8	57/2845	1.7 (1.2–2.1)
	Europe	26	2234/62,854	3.1 (2.5–3.7)
	America	2	62/2178	2.8 (2.1–3.4)
	Africa	3	13/647	1.9 (0.3–3.5)
	Oceania	3	71/1914	3.6 (2.7–4.4)

**Table 2:** Prevalence of *FRSA, FRMRSA* and *FRSSA* based on different countries

Category	Subcategory	No. studies	No. strains	Prevalence (%) (95% CI)
*FRSA*				
Overall	*FRSA*/*S. aureus*	120	7562/157,220	0.5 (4.6–5.4)
Country	Australia	6	796/15,781	4.8 (3.4–6.2)
	Belgium	2	25/663	3.6 (2.2–5.0)
	Canada	5	376/6257	6.3 (3.2–9.3)
	China	4	206/2156	5.3 (0.6–10.0)
	France	7	132/3120	5.0 (3.2–6.8)
	Germany	6	94/2424	4.7 (2.8–6.6)
	India	2	6/173	3.5 (0.7–6.2)
	Iran	3	13/353	3.4 (1.5–5.3)
	Israel	2	8/220	3.6 (1.1–6.0)
	Kuwait	3	93/1370	6.8 (5.4–8.1)
	Malaysia	5	102/1956	5.3 (3.6–6.9)
	Malta	2	374/25	6.5 (4.0–9.1)
	Netherland	2	63/1193	5.2 (4.0–6.5)
	Norway	2	12/136	8.3 (3.7–12.9)
	Poland	2	10/291	3.4 (1.3–5.5)
	Spain	2	5/113	4.2 (0.5–8.0)
	Sweden	2	38/616	5.4 (3.6–7.2)
	Switzerland	2	18/317	5.6 (3.1–8.2)
	Taiwan	4	75/1134	5.9 (1.9–9.9)
	Turkey	9	223/3319	5.8 (3.7–7.9)
	UK	22	4715/105,038	4.8 (4.1–5.5)
	USA	3	26/653	3.7 (2.3–5.2)
*FRMRSA*				
Overall	*FRMRSA*/*S. aureus*	79	2193/94,238	2.6 (2.3–2.9)
Country	Australia	2	33/2881	1.1 (0.8–1.5)
	Canada	4	276/4145	4.5 (0.2–8.9)
	China	4	5/295	1.3 (0.0–2.5)
	France	3	33/2141	1.4 (0.3–2.5)
	Germany	2	11/656	2.0 (0.0–4.5)
	Greece	2	5/142	2.1 (0.3–3.9)
	Iran	4	14/844	1.2 (0.4–1.9)
	Korea	2	19/653	2.1 (0.0–5.0)
	Kuwait	2	91/1427	4.5 (0.1–9.1)
	Malaysia	4	84/1877	4.5 (1.9–7.2)
	Pakistan	2	47/691	4.9 (0.2–12.7)
	Poland	2	8/338	2.0 (0.5–3.5)
	Taiwan	2	2/124	0.9 (0.0–2.1)
	Turkey	7	77/1324	5.3 (2.7–7.9)
	UK	10	1167/67,758	1.4 (1.0–1.9)
	USA	3	26/653	3.7 (2.3–5.2)
*FRMSSA*				
Overall	*FRMRSA*/*MRSA*	72	3211/50,078	5.8 (5.0–6.6)
Country	Australia	3	117/1470	7.7 (1.1–14.3)
	Canada	4	269/3477	6.4 (4.1–8.8)
	China	3	4/126	2.9 (0.0–5.8)
	France	2	74/1437	7.8 (0.0–19.0)
	Germany	2	27/253	10.3 (6.5–14.0)
	Iran	3	10/253	3.5 (1.2–5.7)
	Kuwait	3	847/7002	9.3 (3.6–15.1)
	Malaysia	4	84/1642	4.8 (3.8–5.9)
	Pakistan	2	47/583	6.6 (4.6–8.6)
	Serbia	2	6/144	3.6 (0.6–6.7)
	Taiwan	3	99/716	8.6 (0.4–16.7)
	Turkey	5	49/796	5.6 (2.7–8.6)
	UK	16	1374/27,138	5.1 (4.1–6.1)
	USA	3	26/653	3.7 (2.3–5.2)
*FRMSSA*				
Overall	*FRMSSA*/*S. aureus*	9	939/11,992	6.7 (5.4–7.9)
Country	UK	4	906/11,265	8.0 (7.3–8.8)
Overall	*FRMSSA*/*MSSA*	42	2437/70,438	3.0 (2.6–3.5)
Country	Australia	2	60/1734	3.4 (2.6–4.3)
	Canada	2	62/2178	3.6 (0.6–6.6)
	China	3	37/2203	1.5 (1.0–2.1)
	Germany	3	22/674	2.9 (1.7–4.2)
	Malaysia	2	10/348	2.2 (0.0–5.1)
	Turkey	4	10/382	1.8 (0.5–3.1)
	UK	13	2184/61,050	3.5 (2.8–4.2)

### The prevalence of FRSA, FRMRSA and FRMSSA in different study periods

To determine the longitudinal changes in the prevalence of FRSA, FRMRSA, and FRMSSA across recent years, we designed subgroups across three periods (before 2000, 2000–2009, and 2010–2020) (Tables 1, 2). As shown in Tables 1 and 2, the incidence rate of FRSA and FRMRSA strains gradually increased from 4.0% (95% CI 3.4–4.5) of 3478/90,563 *S. aureus* isolates and 1.4% (95% CI 1.1–1.8) of 547/39,499 MRSA isolates before 2000 to 5.6% (95% CI 4.9–6.2) of 2834/46,134 isolates and 2.9% (95% CI 2.3–3.4) of 1094/41,157 isolates in 2000–2009, reaching 5.2% (95% CI 4.4–6.1) of 1250/20,523 *S. aureus* isolates and 3.2% (95% CI 2.3–4.1) of 552/13,582 MRSA isolates in 2010–2020, respectively. The changes in FRSA, and FRMRSA prevalence and also the changes in FRMSSA prevalence in all three periods are shown in Tables 1 and 2.

### The prevalence of FRSA, FRMRSA and FRMSSA in different regions of the world

Prevalence of FRSA, FRMRSA, and FRMSSA based on geographic area in the subgroup analysis are shown in Tables 1 and 2. As can be seen, the frequency of FRSA in Asia [5.6% (95% CI 4.6–6.6)] is 1.20 and 1.12-fold higher than in Europe [4.7% (95% CI 4.3–5.2)] or Oceania [5.0% (95% CI 3.7–6.3)], respectively. The prevalence of FRSA is almost the same in Asia, America, and Africa. Also, the frequency of FRMRSA in Asia [3.0% (95% CI 2.1–4.10)] is 1.57 and 2.72-fold higher than in Europe [1.9% (95% CI 1.5–2.2)] and Oceania [1.1% (95% CI 0.8–1.5)], respectively. It is noteworthy that the prevalence of FRMRSA in Africa [6.8% (95% CI 3.6–9.9)] and America [4.3% (95% CI 1.5–7.2)] is higher than for other continents.

## Discussion

The emergence of resistance to FA among *S. aureus* isolates has become a matter of concern in many different countries which makes it a threat to public health [153]. According to the evidence, the prevalence rate of FRSA strains differs in various geographic regions and/or patients population. In this systematic review, we noted a low prevalence of resistance to FA in 0.5% [(95% CI) 4.6–5.4] of *S. aureus* isolates reflecting improved infection control precautions and effectiveness of continued surveillance of *S. aureus* infections [14, 153, 234]. The present systematic review illustrated a higher prevalence of FRSA in Asia (5.6%) as compared to other continents. This is of serious concern reflecting inappropriate unrestricted policies and use of FA would similarly be higher in Asian countries [235, 236]. This phenomenon is related to easy access to antibiotics without prescription, paucity of suitable alternatives to FA for topical administration and cheap antibiotics, in these areas [153, 235]. Although it is difficult to recommend completely outlawing use of FA, restricted use of this antibiotic in both community and health care settings and in combination with other antibiotics is highly recommended [234, 237]. It is worth noting that the incidence rate of FRSA strains gradually increased from 4.0% before 2000 to 5.2% in 2010–2020. It seems that this increasing rate is directly linked to the increase in *S. aureus* infections and a shift in antibiotic pressures [14]. The present analyses exhibited a higher prevalence of FRMRSA (5.8%) compared to FRMSSA (3.0%). It is well**-**documented that MRSA isolates exhibited a high prevalence of multi-resistance towards antibiotics of different classes compare to MSSA strains which could limit the choices available for the control of MRSA infections. However, the use of FA outside the hospital is still not justified [236–238]. Furthermore, clinician’ and patient’ education is an important aspect promoting the appropriate use and prescription of FA together with close monitoring of antibiotic susceptibility patterns and use of this antibiotic in combination with other drugs to prevent further emergence of these strains. However, our analysis suggests that both MRSA and MSSA strains must be evaluated routinely in terms of resistance to FA. The current systematic review illustrated a high prevalence of FRMRSA in Africa (6.8%) and America (4.3%) compared to other continents. Although there is wide diversity among MRSA molecular types colonizing and infecting the population in different parts of the world, the higher relative prevalence of FRMRSA highlight the need for clinician’s awareness in administration and use of FA in community and hospital setting in Africa and America. It is well known that too many prescription guidelines are according to the outmoded data gained from observational studies of a small size. Also, it must be borne in mind that there is limited understanding of FA resistance at the epidemiological, clinical, and genetic level [239–241]. Moreover, infection control efforts as a main framework and strategy are crucial to decline the emergence and prevalence of FRMRSA strains [14, 237, 240]. One potential explanation for the higher prevalence of FRMRSA in America (4.3%) could be related to phenotypic methods used and breakpoint values applied for the screening and detection of FRMRSA. There were some drawbacks in the current review. Only published scientific studies were considered for the present meta-analysis and potential publication bias had to be considered. Secondly, we aimed to investigate the prevalence of FRSA, FRMRSA, and FRMSSA in all countries. Since many countries had no record of the prevalence of these strains, we were not able to reach this goal. The prevalence of rampant bacteria in patients with *S. aureus* infection disease is not well established in many countries. So, the prevalence of these FRSA, FRMRSA, and FRMSSA in patients with *S. aureus* infection should be investigated in every country to gather comprehensive information.

## Conclusion

This meta-analysis depicted trends towards an increasing incidence of FRSA, FRMSSA, and FRMRSA. The findings highlight the need for the implementation of (ongoing) surveillance, antibiotic stewardship measures to mitigate the emergence and spread of FRSA, gathering epidemiological data to understand the peculiarities of the epidemiology, medical burden and risk factors related to FRSA, harmonized guidelines for infection control, education of clinicians on the proper prescribing of FA, and development of strategies for monitoring the effects of FA use.

## Supplementary Information

**Additional file 1: Tables S1–S3**. Characteristics of included studies.
