# Supplementary material for: The global prevalence of fusidic acid resistance in clinical isolates of Staphylococcus aureus: a systematic review and meta-analysis
Source: Antimicrob Resist Infect Control. 2021 May 1;10:75. doi: 10.1186/s13756-021-00943-6 (PMC8088720; doi:10.1186/s13756-021-00943-6)
Supplement: Supplementary file 1 — Additional file 1: Tables S1–S3. Characteristics of included studies. [file 13756_2021_943_MOESM1_ESM.docx]

Table S1. Characteristics of included studies which studied FRSA.

| **First author** | **Time of study** | **Published time** | **Country** | **Continent** | **No. *S. aureus*** | **No. FRSA** | **FRSA Frequency (%)** | **Source of isolate** | **Detection methods** | **Ref** |
| --- | --- | --- | --- | --- | --- | --- | --- | --- | --- | --- |
| Nagarajan | 2011 | 2012 | India | Asia | 54 | 2 | 2/54 (3.7) | skin infections | disk diffusion method-multiplex-PCR | ([1](#_ENREF_1)) |
| Akpabie | 2003 | 2004 | France | Europe | 213 | 3 | 3/213 (1.4) | nr | disk diffusion test | ([2](#_ENREF_2)) |
| Österlund | 1990–2001 | 2002 | Sweden | Europe | 10519 | 1500 | 1500/10519 (14.2) | swab skin | PFGE-Susceptibility testing | ([3](#_ENREF_3)) |
| Larsen | 1993 - 2004 | 2007 | Denmark | Europe | 294 | 277 | 277/294 (94.2) | nr | agar dilution | ([4](#_ENREF_4)) |
| Sarkar | 2013-2014 | 2016 | Saudi Arabia | Asia | 32 | 4 | 4/32 (12.5) | nasal swab sample | Antimicrobial susceptibility testing-DNA microarray | ([5](#_ENREF_5)) |
| [Senok](https://www.liebertpub.com/doi/10.1089/mdr.2019.0318) | nr | 2019 | UAE | Asia | 29 | 4 | 4/29 (13.7) | Nasal swabs | nr | ([6](#_ENREF_6)) |
| Senok | 2017 | 2019 | Saudi Arabia | Asia | 125 | 54 | 54/125 (43.2) | nr | antibiotic susceptibility testing-DNA microarray | ([7](#_ENREF_7)) |
| Ahmadi | 2017-2018 | 2019 | Iran | Asia | 181 | 1 | 1/181 (0.5) | anterior nares | Agar disk diffusion method | ([8](#_ENREF_8)) |
| Nawaz | nr | 2016 | Pakistan | Asia | 103 | 55 | 55/103 (53.3) | surgical wound specimens | Kirby-Bauer diffusion method. PCR | ([9](#_ENREF_9)) |
| Akinkunmi | 2014 | 2014 | Nigeria | Africa | 23 | 9 | 9/23 (39.1) | wound | disk diffusion | ([10](#_ENREF_10)) |
| Aktas | nr | 2017 | turkey | Europe | 25 | 18 | 18/25 (72.0) | nr | microbroth dilution assay | ([11](#_ENREF_11)) |
| [Sweih](https://www.ncbi.nlm.nih.gov/pubmed/?term=Al%20Sweih%20N%5BAuthor%5D&cauthor=true&cauthor_uid=16433190) | nr | 2005 | Kuwait | Asia | 1983 | 922 | 922/1983 (46.4) | different sources | E. test, disk diffusion and Vitek | ([12](#_ENREF_12)) |
| [Abouelfetouh](https://www.researchgate.net/profile/Alaa_Abouelfetouh) | 2011 | 2016 | Egypt | Africa | 81 | 26 | 26/81 (32.0) | pus,bloodmsputum,urine,BA lavage | PCR | ([13](#_ENREF_13)) |
| Aldasouqi | 2015 | 2019 | Jordan | Asia | 113 | 36 | 36/113 (31.8) | urine,blood,tissue,respiratory secretion | disk diffusion | ([14](#_ENREF_14)) |
| Somily | 2005-2006 | 2012 | Saudi Arabia | Asia | 122 | 49 | 49/122 (40.1) | soft tissue including pus, joint fluid, and blood | disk diffusion, E. test | ([15](#_ENREF_15)) |
| Hasani | nr | 2013 | Iran | Asia | 150 | 7 | 7/150 (4.6) | blood,urine, postoperative wound, synovial fluid, sputum | E. test/PCR | ([16](#_ENREF_16)) |
| Al-Talib | 2008 | 2015 | Malaysia | Asia | 158 | 20 | 20/158 (12.6) | blood, cerebrospinal fluid, respiratory tract, eye, pus and wound, urine, genital specimens, environment and other sites | Antibiotic susceptibility testing | ([17](#_ENREF_17)) |
| Aqel | 2011-2012 | 2015 | Jordan | Asia | 56 | 3 | 3/56 (5.3) | nasal swabs | PCR | ([18](#_ENREF_18)) |
| Cabrera | 2010 | 2016 | Canada | America | 3036 | 246 | 246/3036 (8.1) | Bloodstream infections, skin/soft tissue, respiratory, surgical site, urine and other sites. | PFGE | ([19](#_ENREF_19)) |
| [Vindel](https://www.researchgate.net/scientific-contributions/39208791_Ana_Vindel) | 2004-2012 | 2014 | Spain | Europe | 8326 | 166 | 166/8326 (1.9) | wound and other areas | PCR | ([20](#_ENREF_20)) |
| Doudoulakakis | 2013-2016 | 2017 | Greece | Europe | 102 | 97 | 97/102 (95.0) | skin | PCR-molecular typing | ([21](#_ENREF_21)) |
| Shore | 1971-2004 | 2012 | Germany | Europe | 175 | 21 | 21/175 (12.0) | nr | multiplex PCR-DNA microarray analysis-spa typing | ([22](#_ENREF_22)) |
| Atmaca | nr | 2001 | Turkey | Europe | 103 | 12 | 12/103 (11.6) | nr | disk diffusion | ([23](#_ENREF_23)) |
| Baek | 2009-2011 | 2012 | Korea | Asia | 482 | 221 | 221/482 (45.8) | Skin | nr | ([24](#_ENREF_24)) |
| Ahmad | nr | 2014 | Pakistan | Asia | 54 | 9 | 9/54 (16.6) | pus | Susceptibility test for antimicrobial agent-PFGE method-PCR | ([25](#_ENREF_25)) |
| Bauer | 1999-2007 | 2009 | Austria | Europe | 2542 | 81 | 81/2542 (3.1) | skin,wound,pus,abscess,nasal,miscellaneous,endotracheal | disk diffusion | ([26](#_ENREF_26)) |
| Berktold | 2005-2010 | 2012 | Australia | Oceania | 650 | 84 | 84/650 (12.9) | nr | nr | ([27](#_ENREF_27)) |
| Bernard | 2003-2004 | 2007 | France | Europe | 205 | 9 | 9/205 (4.3) | nr | Antimicrobial susceptibility | ([28](#_ENREF_28)) |
| Bessa | nr | 2016 | Brazil | America | 100 | 6 | 6/100 (6.0) | the anterior nose cavity and an eczema plaque | disk diffusion,MIC by E. test | ([29](#_ENREF_29)) |
| Boswihi | 1992-2010 | 2016 | Kuwait | Asia | 400 | 214 | 214/400 (53.5) | nr | antibiogram | ([30](#_ENREF_30)) |
| Brosnikoff | nr | 2009 | Canada | America | 602 | 44 | 44/602 (7.3) | clinical strains | disk diffusion | ([31](#_ENREF_31)) |
| [Budimir](https://europepmc.org/search?query=AUTH:%22Ana%20Budimir%22) | 2001-2007 | 2016 | Croatia | Europe | 46 | 1 | 1/46 (2.1) | Blood | PFGE | ([32](#_ENREF_32)) |
| Budri | 2014-2016 | 2018 | Ireland | Europe | 137 | 33 | 33/137 (24.0) | nasal | E. test | ([33](#_ENREF_33)) |
| Horner | nr | 2012 | England | Europe | 888 | 118 | 118/888 (13.2) | nasal | disk susceptibility testing method | ([34](#_ENREF_34)) |
| Quentin | 1998 | 2001 | France | Europe | 747 | 104 | 104/747 (13.9) | pus | disk diffusion method | ([35](#_ENREF_35)) |
| Claesson | 2008 | 2009 | Sweden | Europe | 453 | 33 | 33/453 (7.2) | skin and soft tissue | E. test | ([36](#_ENREF_36)) |
| Castanheira | 2008-2009 | 2010 | Poland | Europe | 66 | 1 | 1/66 (1.5) | nr | broth microdilution,PCR,PFGE | ([37](#_ENREF_37)) |
| Castanheira | 2008-2009 | 2010 | Israel | Europe | 70 | 2 | 2/70 (2.8) | nr | broth microdilution,PCR,PFGE | ([37](#_ENREF_37)) |
| Castanheira | 2008-2009 | 2010 | Spain | Europe | 208 | 3 | 3/208 (1.4) | nr | broth microdilution,PCR,PFGE | ([37](#_ENREF_37)) |
| Castanheira | 2008-2009 | 2010 | Switzerland | Europe | 59 | 4 | 4/59 (6.7) | nr | broth microdilution,PCR,PFGE | ([37](#_ENREF_37)) |
| Castanheira | 2008-2009 | 2010 | Italy | Europe | 147 | 4 | 4/147 (2.7) | nr | broth microdilution,PCR,PFGE | ([37](#_ENREF_37)) |
| Castanheira | 2008-2009 | 2010 | Sweden | Europe | 163 | 5 | 5/163 (3.0) | nr | broth microdilution,PCR,PFGE | ([37](#_ENREF_37)) |
| Castanheira | 2008-2009 | 2010 | Belgium | Europe | 93 | 6 | 6/93 (6.4) | nr | broth microdilution,PCR,PFGE | ([37](#_ENREF_37)) |
| Castanheira | 2007-2008 | 2010 | Australia | [Oceania](https://www.internetworldstats.com/europa2.htm) | 100 | 7 | 7/100 (7.0) | blood-skin-respiratory infection | broth microdilution-pcr | ([38](#_ENREF_38)) |
| Castanheira | 2007-2008 | 2010 | Canada | America | 100 | 7 | 7/100 (7.0) | blood-skin-respiratory infection | broth microdilution-pcr | ([38](#_ENREF_38)) |
| Castanheira | 2008-2009 | 2010 | Turkey | Europe | 128 | 8 | 8/128 (6.2) | nr | broth microdilution,PCR,PFGE | ([37](#_ENREF_37)) |
| Castanheira | 2007-2008 | 2010 | USA | America | 3666 | 11 | 11/3666 (0.3) | blood-skin-respiratory infection | broth microdilution-pcr | ([38](#_ENREF_38)) |
| Castanheira | 2008-2009 | 2010 | UK | Europe | 289 | 34 | 34/289 (11.7) | nr | broth microdilution,PCR,PFGE | ([37](#_ENREF_37)) |
| Castanheira | 2008-2009 | 2010 | Ireland | Europe | 241 | 48 | 48/241 (19.9) | nr | broth microdilution,PCR,PFGE | ([37](#_ENREF_37)) |
| Castanheira | 2008-2009 | 2010 | Greece | Europe | 242 | 127 | 127/242 (52.4) | nr | broth microdilution,PCR,PFGE | ([37](#_ENREF_37)) |
| Castanheira | 2008-2009 | 2010 | Germany | Europe | 453 | 13 | 13/453 (2.8) | nr | broth microdilution,PCR,PFGE | ([37](#_ENREF_37)) |
| Castanheira | 2008-2009 | 2010 | France | Europe | 541 | 35 | 35/541 (6.4) | nr | broth microdilution,PCR,PFGE | ([37](#_ENREF_37)) |
| Cetinkol | 2018 | 2018 | Turkey | Europe | 2018 | 147 | 147/2018 (7.2) | wounds, blood, aspirate, abscess, and catheters | Antibiotic susceptibility testing | ([39](#_ENREF_39)) |
| Chen | 2002-2007 | 2010 | Taiwan | Asia | 71 | 71 | 71/71 (100.0) | Pus,Blood ,Urine ,Sputum,Miscellaneous (tissue,HVS and implants) | broth microdilution/PCR | ([40](#_ENREF_40)) |
| Chen | 2007 - 2008 | 2011 | Taiwan | Asia | 34 | 25 | 25/34 (73.5) | Pus,Blood ,Urine ,Sputum,Miscellaneous (tissue,HVS and implants) | broth microdilution/PCR | ([41](#_ENREF_41)) |
| Chuamuangphan | 2012 | 2013 | Korea | Asia | 156 | 1 | 1/156 (0.6) | Blood | disk diffusion method | ([42](#_ENREF_42)) |
| Cirkovic | nr | 2010 | Serbia | Asia | 21 | 2 | 2/21 (9.5) | skin and soft tissue | MIC | ([43](#_ENREF_43)) |
| Yeung | 2006 | 2010 | Hong Kong | Asia | 142 | 4 | 4/142 (2.8) | nr | Antibiotic susceptibility testing-Molecular typing | ([44](#_ENREF_44)) |
| Conceicao | nr | 2019 | Portugal | Europe | 43 | 9 | 9/43 (20.9) | nasal | disk diffusion method | ([45](#_ENREF_45)) |
| Coskun | 2011-2014 | 2019 | Turkey | Europe | 84 | 36 | 36/84 (42.8) | wound, blood and urine | disk diffusion method, E. test | ([46](#_ENREF_46)) |
| Sahm | nr | 2013 | USA | America | 51 | 49 | 49/51 (96.0) | bloodstream,respiratory tract,wound,skin | disk diffusion | ([47](#_ENREF_47)) |
| RX | nr | 2019 | Australia | Oceania | 215 | 8 | 8/215 (3.7) | skin and superficial soft tissue | Susceptibility test methods | ([48](#_ENREF_48)) |
| Farrell | 2014 | 2016 | USA | America | 1804 | 4 | 4/1804 (0.2) | nr | broth dilution | ([49](#_ENREF_49)) |
| Decousser | 2011-2012 | 2015 | France | Europe | 367 | 50 | 50/367 (13.6) | from bacteraemia and osteoarticular infections | broth microdilution | ([50](#_ENREF_50)) |
| Demir | 2007-2008 | 2012 | Turkey | Europe | 242 | 6 | 6/242 (2.4) | wound | Kirby–Bauer disk diffusion | ([51](#_ENREF_51)) |
| Heijer | 2010-2011 | 2014 | Austria | Europe | 541 | 5 | 5/541 (0.9) | nasal swabs | MIC/PCR | ([52](#_ENREF_52)) |
| Heijer | 2010-2011 | 2014 | Belgium | Europe | 570 | 19 | 19/570 (3.3) | nasal swabs | MIC/PCR | ([52](#_ENREF_52)) |
| Heijer | 2010-2011 | 2014 | Croatia | Europe | 740 | 1 | 1/740 (0.1) | nasal swabs | MIC/PCR | ([52](#_ENREF_52)) |
| Heijer | 2010-2011 | 2014 | France | Europe | 858 | 27 | 27/585 (3.1) | nasal swabs | MIC/PCR | ([52](#_ENREF_52)) |
| Heijer | 2010-2011 | 2014 | Hungary | Europe | 531 | 1 | 1/531 (0.1) | nasal swabs | MIC/PCR | ([52](#_ENREF_52)) |
| Heijer | 2010-2011 | 2014 | Spain | Europe | 756 | 7 | 7/756 (0.9) | nasal swabs | MIC/PCR | ([52](#_ENREF_52)) |
| Heijer | 2010-2011 | 2014 | Sweden | Europe | 955 | 18 | 18/955 (1.8) | nasal swabs | MIC/PCR | ([52](#_ENREF_52)) |
| Heijer | 2010-2011 | 2014 | UK | Europe | 799 | 62 | 62/799 (7.7) | nasal swabs | MIC/PCR | ([52](#_ENREF_52)) |
| Heijer | 2010-2011 | 2014 | Netherlands | Europe | 1064 | 54 | 54/1064 (5.0) | nasal swabs | MIC/PCR | ([52](#_ENREF_52)) |
| Denis | 2002-2004 | 2005 | Belgian | Europe | 41 | 23 | 23/41 (56.0) | skin or soft tissue specimens from upper limbs , trunk, lower limbs, throat swab, blood and peritoneal fluid | disk diffusion | ([53](#_ENREF_53)) |
| Ghaith | 2019 | 2019 | Egypt | Africa | 250 | 50 | 50/250 (20.0) | wound and skin infections | PCR | ([54](#_ENREF_54)) |
| [Anastasiou](https://www.ijidonline.com/article/S1201-9712(11)60068-4/fulltext) | 2008-2010 | 2011 | Greece | Europe | 140 | 3 | 3/140 (2.1) | pus,blood,urine,trauma | agar dilution | ([55](#_ENREF_55)) |
| Lindberg | 2003 | 2004 | Sweden | Europe | 116 | 1 | 1/116 (0.8) | commensal intestinal microflora | Antibiotic susceptibility testing-PCR | ([56](#_ENREF_56)) |
| Udo | 2011 | 2012 | Kuwait | Asia | 21 | 2 | 2/21 (9.5) | 20 neonates | antibiogram, PFGE | ([57](#_ENREF_57)) |
| Udo | 2018 | 2020 | Kuwait | Asia | 97 | 97 | 97/97 (100.0) | nr | MIC / PCR | ([58](#_ENREF_58)) |
| Champion | 2008-2010 | 2013 | USA | America | 277 | 8 | 8/277 (2.8) | Respiratory secretions | multiplex PCR, E-tests | ([59](#_ENREF_59)) |
| Udo | 1996-2001 | 2006 | Kuwait | Asia | 88 | 74 | 74/88 (84.0) | skin swabs | disk diffusion | ([60](#_ENREF_60)) |
| Udo | 2001 | 2006 | Kuwait | Asia | 21 | 18 | 18/21 (85.7) | skin swabs | disk diffusion | ([60](#_ENREF_60)) |
| Udo | nr | 2010 | Kuwait | Asia | 135 | 57 | 57/135 (42.2) | wound swabs | nr | ([61](#_ENREF_61)) |
| Udo | 1994-2004 | 2006 | Kuwait | Asia | 5644 | 756 | 756/5644 (13.3) | abscesses, nasal swabs, ear swabs | nr | ([62](#_ENREF_62)) |
| Udo | 2011 | 2013 | Kuwait | Asia | 22 | 2 | 2/22 (9.0) | blood samples, groin | nr | ([63](#_ENREF_63)) |
| Udo | 2006-2011 | 2017 | Kuwait | Asia | 103 | 17 | 17/103 (16.5) | throat swabs, urine samples, skin | nr | ([64](#_ENREF_64)) |
| Udo | 2011–2015 | 2017 | Kuwait | Asia | 6922 | 2,858 | 2,858/6922 (41.2) | swabs, tracheal aspirates, high vaginal swabs, sputum and eye swab | nr | ([65](#_ENREF_65)) |
| Udo | 2005-2006 | 2010 | Kuwait | Asia | 135 | 57 | 57/135 (42.2) | nr | disk diffusion method, PFGE | ([66](#_ENREF_66)) |
| Udo | nr | 2008 | Bahrain | Asia | 53 | 49 | 49/53 (92.4) | nr | disk diffusion | ([67](#_ENREF_67)) |
| Edslev | nr | 2018 | Denmark | Europe | 138 | 57 | 57/138 (41.3) | umbilical stump swabs, nasal swabs, blood, axilla, eye swabs and catheter tips | PFGE, MLST | ([68](#_ENREF_68)) |
| Egyir | nr | 2013 | Ghana | Africa | 105 | 13 | 13/105 (12.3) | nr | Antibiotic susceptibility testing, PCR,MLST | ([69](#_ENREF_69)) |
| Champion | nr | 2011 | USA | America | 282 | 14 | 14/282 (4.9) | skin and soft tissues samples, umbilical cord stumps, nasal swabs, eye swabs, | Susceptibility to Antimicrobial Agents | ([70](#_ENREF_70)) |
| [Scicluna](https://www.researchgate.net/profile/Elizabeth_Scicluna) | 2008-2009 | 2009 | Malta | Europe | 45 | 2 | 2/45 (4.4) | nr | MIC | ([71](#_ENREF_71)) |
| El-Zimaity | 1997-2001 | 2004 | UK | Europe | 1797 | 119 | 119/1797 (6.6) | hospital patients | nr | ([72](#_ENREF_72)) |
| Brown | 1998 | 2002 | UK | Europe | 2542 | 127 | 127/2542 ()4.9 | lesional skin,nonlesional skin,anterior nares | disk diffusion | ([73](#_ENREF_73)) |
| Brown | 2000 | 2002 | UK | Europe | 2893 | 180 | 180/2893 (6.2) | nasal | disk diffusion | ([73](#_ENREF_73)) |
| Brown | 1999 | 2002 | UK | Europe | 2883 | 185 | 185/2883 (6.4) | CF patient | nr | ([73](#_ENREF_73)) |
| Brown | 2001 | 2002 | UK | Europe | 2947 | 212 | 212/2947 (7.1) | nr | hybridization | ([73](#_ENREF_73)) |
| Cercenado | 2004 to 2007 | 2008 | Spain | Europe | 13 | 1 | 1/13 (7.6) | skin swabs | antibiogram | ([74](#_ENREF_74)) |
| Erdenizmenli | 1999-2001 | 2004 | Turkey | Europe | 91 | 17 | 17/91 (18.6) | nr | nr | ([75](#_ENREF_75)) |
| Hanif | nr | 2019 | Pakistan | Asia | 250 | 156 | 156/250 (62.4) | nr | nr | ([76](#_ENREF_76)) |
| Khan | 2012 | 2018 | Pakistan | Asia | 64 | 13 | 13/64 (20.3) | nr | nr | ([77](#_ENREF_77)) |
| McLaws | 2003-2005 | 2011 | Denmark | Europe | 1639 | 291 | 291/1639 (17.7) | nr | nr | ([78](#_ENREF_78)) |
| Idrees | 2005-2007 | 2009 | Pakistan | Asia | 501 | 45 | 45/501 (8.9) | skin and soft tissue infections, otitis, and bacteremia | E-test, PFGE, MLST | ([79](#_ENREF_79)) |
| Fangyou | 2012-2013 | 2015 | China | Asia | 392 | 56 | 56/392 (14.2) | blood samples | nr | ([80](#_ENREF_80)) |
| Rahimi | 2013 | 2016 | Iran | Asia | 491 | 4 | 4/491 (0.8) | urine, pus, blood, tissue, wound and ear swabs | disk diffusion | ([81](#_ENREF_81)) |
| Bari | 2013 | 2015 | Pakistan | Asia | 957 | 690 | 690/957 (72.1) | floor, walls, air and inanimate objects | PCR | ([82](#_ENREF_82)) |
| Fong | nr | 2018 | Malaysia | Asia | 56 | 19 | 19/56 (33.9) | nr | disk diffusion assay-PFGE, MLST | ([83](#_ENREF_83)) |
| Schaefer | 1997-1998 | 2001 | Switzerland | Europe | 19 | 10 | 10/19 (52.6) | skin and soft tissue specimens | disk diffusion | ([84](#_ENREF_84)) |
| [Randrianirina](https://www.ncbi.nlm.nih.gov/pubmed/?term=Randrianirina%20F%5BAuthor%5D&cauthor=true&cauthor_uid=17521424) | 2001-2005 | 2007 | Madagascar | Africa | 529 | 52 | 52/529 (9.8) | Sputum, pus, catheter, and exudates | disk diffusion-MIC | ([85](#_ENREF_85)) |
| Frickmann | nr | 2019 | Germany | Europe | 1040 | 24 | 24/1040 (2.3) | wound,urine, blood, sputum, CSF, earand eye | PCR | ([86](#_ENREF_86)) |
| Cartolano | 2000 | 2004 | France | Europe | 1070 | 24 | 24/1070 (2.2) | nr | nr | ([87](#_ENREF_87)) |
| Coombs | 2011 | 2013 | Australia | Oceania | 2357 | 81 | 81/2357 (3.4) | Pus,Blood ,Urine ,Sputum,Miscellaneous | broth microdilution/PCR | ([88](#_ENREF_88)) |
| Gilani | nr | 2005 | UK | Europe | 18 | 7 | 7/18 (38.8) | Nasal | disk diffusion | ([89](#_ENREF_89)) |
| Gordon | nr | 2014 | UK | Europe | 501 | 43 | 43/501 (8.5) | bacterial keratitis-chronic ulcerations,patients with a corneal ulceration | Antibiotic susceptibility testing | ([90](#_ENREF_90)) |
| Gosbell | 1990-1999 | 2001 | Australia | Oceania | 12909 | 684 | 684/12909 (5.2) | hospitalized patient | disk diffusion | ([91](#_ENREF_91)) |
| Gubbay | 2001-2002 | 2008 | Australia | Oceania | 100 | 11 | 11/100 (11.0) | blood | VITEK 2 | ([92](#_ENREF_92)) |
| Belabbès | 2000 | 2001 | Morocco | Africa | 189 | 87 | 87/189 (46.0) | Skin and soft tissue infection specimens | vitek2 | ([93](#_ENREF_93)) |
| Harkins | nr | 2016 | UK | Europe | 49 | 6 | 6/49 (12.2) | affected skin, anterior nares | standard streak method | ([94](#_ENREF_94)) |
| Harkins, | nr | 2016 | UK | Europe | 50 | 12 | 12/50 (24.0) | nasal swab, blood | broth dilution | ([94](#_ENREF_94)) |
| Heng | nr | 2013 | Singapore | Asia | 322 | 37 | 37/322 (11.4) | serious soft tissue infections | E-test, PCR, phage typing and PFGE | ([95](#_ENREF_95)) |
| Harastani | 2000-2011 | 2014 | Lebanon | Asia | 94 | 34 | 3494 (36.1) | skin/soft tissue, four bone andjoint, four surgical site infections, three pneumonia, eight other types, and represented colonisation | disk diffusion testing -PFGE-PCR | ([96](#_ENREF_96)) |
| Huang | nr | 2013 | China | Asia | 116 | 4 | 4/116 (3.4) | nosocomial | disk diffusion method | ([97](#_ENREF_97)) |
| Al Balawi | 2014 | 2017 | Arabia | Asia | 54 | 16 | 16/54 (29.6) | nosocomial | disk diffusion method | ([98](#_ENREF_98)) |
| Błażewicz | 2014-2015 | 2017 | Poland | Europe | 148 | 30 | 30/148 (20.2) | nasal | MIC | ([99](#_ENREF_99)) |
| Wang | 2004 | 2017 | Taiwan | Asia | 466 | 15 | 15/466 (3.2) | eczema site | MIC | ([100](#_ENREF_100)) |
| Wang | 2008 | 2017 | Taiwan | Asia | 475 | 51 | 51/475 (10.7) | skin | disk diffusion test | ([100](#_ENREF_100)) |
| Wang | 2012 | 2017 | Taiwan | Asia | 476 | 86 | 86/476 (18.0) | nr | Multiplex PCR, PFGE | ([100](#_ENREF_100)) |
| [Li](https://www.researchgate.net/scientific-contributions/2095188041_J_Li) | nr | 2014 | China | Asia | 135 | 1 | 1/135 (0.7) | abscess or wound exudates, sputum, blood and urine | disk diffusion method,PCR, PFGE | ([101](#_ENREF_101)) |
| Jappe | 2003-2005 | 2008 | Germany | Europe | 130 | 11 | 11/130 (8.4) | wound sepsis samples, wound infections | Antibiotic Susceptibility Testing | ([102](#_ENREF_102)) |
| Jones | 1997-2006 | 2010 | USA | America | 778 | 14 | 14/778 (1.7) | skin and nasal swabs | MIC | ([103](#_ENREF_103)) |
| Jorgen | 2001 | 2007 | Norway | Europe | 86 | 6 | 6/86 (6.9) | nr | nr | ([104](#_ENREF_104)) |
| Jorgen | 2004 | 2007 | Norway | Europe | 50 | 6 | 6/50 (12.0) | nr | nr | ([104](#_ENREF_104)) |
| Jorgen | 1995-2001 | 2007 | Norway | Europe | 86 | 23 | 23/86 (26.7) | nr | nr | ([104](#_ENREF_104)) |
| Jorgen | 2004 | 2007 | Norway | Europe | 33 | 6 | 6/33 (18.1) | nr | PFGE and Southern blot | ([104](#_ENREF_104)) |
| Hwang | 2000-2001 | 2002 | Taiwan | Asia | 77 | 5 | 5/77 (6.4) | Skin infections | E. test,disk diffusion, broth microdilution | ([105](#_ENREF_105)) |
| Park | 2010-2012 | 2016 | Korea | Asia | 182 | 97 | 97/182 (53.2) | Blood | disk diffusion | ([106](#_ENREF_106)) |
| Karolina Bierowiec | 2013-2014 | 2016 | Poland | Europe | 101 | 4 | 4/101 (3.9) | nr | nr | ([107](#_ENREF_107)) |
| Kedzierska | nr | 2008 | Poland | Europe | 179 | 3 | 3/179 (1.6) | nr | nr | ([108](#_ENREF_108)) |
| Kesah | 1996-1997 | 2003 | Malta | Europe | 10 | 2 | 2/10 (20.0) | chronic otitis media, acute otitis externa and granular myringitis | Kirby–Bauer method | ([109](#_ENREF_109)) |
| Kesah | 1996-1997 | 2003 | Senegal | Africa | 21 | 7 | 7/21 (33.3) | diskharging ears | Kirby-Bauer method | ([109](#_ENREF_109)) |
| Kesah | 1996-1997 | 2003 | Morocco | Africa | 21 | 11 | 11/21 (52.3) | Lesional and nasal specimens | antibiotic susceptibility tests | ([109](#_ENREF_109)) |
| Kesah | 1996-1997 | 2003 | Kenya | Africa | 38 | 22 | 22/38 (57.8) | clinical strains | MIC | ([109](#_ENREF_109)) |
| Kesah | 1996-1997 | 2003 | Nigeria | Africa | 42 | 12 | 12/42 (28.5) | conjunctival sacs, nares , groin , and anus | Antimicrobial susceptibility testing-PCR | ([109](#_ENREF_109)) |
| Kim | 2004-2005 | 2006 | Korea | Asia | 85 | 11 | 11/85 (12.9) | pharyngeal swab,sputum,nasal diskharge,ear swab | disk-diffusion method and the method of serial dilution | ([110](#_ENREF_110)) |
| Kim | nr | 2009 | Korea | Asia | 20 | 18 | 18/20 (90.0) | lesion and nonlesion agar disk-diffusion | agar disk-diffusion method | ([111](#_ENREF_111)) |
| Lim | nr | 2013 | Malaysia | Asia | 162 | 15 | 15/162 (9.2) | sputum samples | nr | ([112](#_ENREF_112)) |
| Klein | 2009–2014 | 2016 | Germany | Europe | 526 | 19 | 19/526 (3.6) | deep throat swab | E. test | ([113](#_ENREF_113)) |
| Bhutia | 2009-2010 | 2012 | India | Asia | 119 | 4 | 4/119 (3.3) | sputum samples | nr | ([114](#_ENREF_114)) |
| Laurent | 2000-2007 | 2009 | France | Europe | 44 | 6 | 6/44 (13.6) | deep throat swab | E. test | ([115](#_ENREF_115)) |
| Lescat | 1993-2002 | 2004 | France | Europe | 33 | 3 | 3/33 (9.0) | sputum samples | nr | ([116](#_ENREF_116)) |
| Livermore | 1990 | 2002 | UK | Europe | 4800 | 2 | 2/4800 (0.0) | deep throat swab | E. test | ([117](#_ENREF_117)) |
| Livermore | 1991 | 2002 | UK | Europe | 4966 | 141 | 141/4966 (2.8) | sputum samples | nr | ([117](#_ENREF_117)) |
| Livermore | 1992 | 2002 | UK | Europe | 5049 | 111 | 111/5049 (2.1) | deep throat swab | E. test | ([117](#_ENREF_117)) |
| Livermore | 1993 | 2002 | UK | Europe | 5741 | 149 | 149/5741 (2.5) | sputum samples | nr | ([117](#_ENREF_117)) |
| Livermore | 1994 | 2002 | UK | Europe | 5960 | 131 | 131/5960 (2.1) | deep throat swab | E. test | ([117](#_ENREF_117)) |
| Livermore | 1995 | 2002 | UK | Europe | 6588 | 171 | 171/6588 (2.5) | sputum samples | nr | ([117](#_ENREF_117)) |
| Livermore | 1996 | 2002 | UK | Europe | 7779 | 280 | 280/7779 (3.5) | deep throat swab | E. test | ([117](#_ENREF_117)) |
| Livermore | 1997 | 2002 | UK | Europe | 9328 | 364 | 364/9328 (3.9) | dermatology inpatients | nr | ([117](#_ENREF_117)) |
| Livermore | 1998 | 2002 | UK | Europe | 9374 | 384 | 384/9374 (4.0) | Eschar Muscle Blood Lung | agar dilution test and E. test | ([117](#_ENREF_117)) |
| Livermore | 1999 | 2002 | UK | Europe | 10813 | 443 | 443/10813 (4.0) | joint fluids /Bone and joint infections | PCR | ([117](#_ENREF_117)) |
| Livermore | 2000 | 2002 | UK | Europe | 11635 | 791 | 791/11635 (6.7) | skin & blood | Kirby-Bauer disk diffusion | ([117](#_ENREF_117)) |
| Livermore | 2001 | 2002 | UK | Europe | 12631 | 770 | 770/12631 (6.0) | Skin infection | disk diffusion | ([117](#_ENREF_117)) |
| Lorette | nr | 2003 | French | Europe | 208 | 21 | 21/208 (10.0) | blood,urine,pus | French standard disk method | ([118](#_ENREF_118)) |
| Louai | 2005 | 2006 | Saudi Arabia | Asia | 110 | 18 | 18/110 (16.3) | blood | nr | ([119](#_ENREF_119)) |
| Louai | 2011 | 2015 | Saudi Arabia | Asia | 93 | 4 | 4/93 (4.3) | blood | nr | ([119](#_ENREF_119)) |
| Shah | 2001 | 2003 | UK | Europe | 48 | 24 | 24/48 (50.0) | blood | nr | ([120](#_ENREF_120)) |
| Alreshidi | nr | 2011 | Malaysia | Asia | 120 | 38 | 38/120 (31.6) | blood | nr | ([121](#_ENREF_121)) |
| [Razeghi](https://www.sciencedirect.com/science/article/pii/S2452014419300536#!) | nr | 2019 | Iran | Asia | 83 | 3 | 3/83 (3.6) | blood | nr | ([122](#_ENREF_122)) |
| Denton | 2003-2004 | 2008 | France | Europe | 205 | 13 | 13/205 (6.3) | blood | nr | ([123](#_ENREF_123)) |
| Denton | 2003-2004 | 2008 | UK | Europe | 221 | 54 | 54/221 (24.4) | blood | nr | ([123](#_ENREF_123)) |
| Denton | 2003-2004 | 2008 | Ireland | Europe | 220 | 69 | 69/220 (31.3) | blood | nr | ([123](#_ENREF_123)) |
| Groome | 2005-2006 | 2012 | South Africa | Africa | 161 | 25 | 25/161 (15.5) | blood | nr | ([124](#_ENREF_124)) |
| [Sun](https://www.researchgate.net/scientific-contributions/2089395898_M_Sun) | nr | 2013 | China | Asia | 60 | 2 | 2/60 (3.3) | blood | nr | ([125](#_ENREF_125)) |
| Margarete | 2005-2006 | 2008 | Germany | Europe | 102 | 26 | 26/102 (25.4) | blood | nr | ([126](#_ENREF_126)) |
| Dinić | nr | 2013 | Serbia | Asia | 1381 | 7 | 7/1381 (0.5) | blood | nr | ([127](#_ENREF_127)) |
| Abdallah | 2005 | 2007 | Egypt | Africa | 50 | 16 | 16/50 (32.0) | skin and soft tissue infections | nr | ([128](#_ENREF_128)) |
| Ellington | 2002-2012 | 2015 | UK | Europe | 23 | 13 | 13/23 (56.5) | skin swab and impetigo | cultures and susceptibility testing | ([129](#_ENREF_129)) |
| Goudarzi | nr | 2020 | Iran | Asia | 120 | 3 | 3/120 (2.5) | skin swab | disk diffusion | ([130](#_ENREF_130)) |
| Memikoǧlu | nr | 2002 | Turkey | Europe | 225 | 6 | 6/225 (2.6) | nr | Kirby-Bauer disk diffusion and broth microdilution | ([131](#_ENREF_131)) |
| Alsterholm | 2004-2008 | 2010 | Sweden | Europe | 171 | 31 | 31/171 (18.1) | various clinical specimens | broth microdilution -disk diffusion method (Kirby-Bauer) | ([132](#_ENREF_132)) |
| Mitra | 2001 | 2009 | UK | Europe | 349 | 53 | 53/349 (15.1) | skin lesion | microdilution broth method | ([133](#_ENREF_133)) |
| Mitra | 2004 | 2009 | UK | Europe | 604 | 104 | 104/604 (17.2) | skin lesion | microdilution broth method | ([133](#_ENREF_133)) |
| Elazhari | 2007-2008 | 2010 | Morocco | Africa | 140 | 18 | 18/140 (12.8) | skin lesion | microdilution broth method | ([134](#_ENREF_134)) |
| Shahmohammadi | nr | 2016 | Iran | Asia | 183 | 36 | 36/183 (19.6) | clinical strains | disk diffusion testing-PCR | ([135](#_ENREF_135)) |
| Khemiri | 2013 | 2017 | Libya | Africa | 32 | 32 | 32/32 (100.0) | skin and soft tissue | disk diffusion method (Kirby-Bauer) | ([136](#_ENREF_136)) |
| Morrissey | nr | 2004 | UK | Europe | 158 | 8 | 8/158 (5.0) | skin swabs | microdilution breakpoint method (VITEK 2 ) or the E. test | ([137](#_ENREF_137)) |
| Nejma | 2003-2005 | 2008 | Tunisia | Africa | 64 | 2 | 2/64 (3.1) | anterior nares | disk diffusion method (Kirby-Bauer) | ([138](#_ENREF_138)) |
| Sohail | 2012-2016 | 2018 | Pakistan | Asia | 344 | 72 | 72/344 (20.9) | skin swabs | disk diffusion | ([139](#_ENREF_139)) |
| Woodford | nr | 2008 | UK | Europe | 664 | 336 | 336/664 (50.6) | Blood | antimicrobial susceptibility test-PCR | ([140](#_ENREF_140)) |
| Nergiz | 2001-2011 | 2012 | turkey | Europe | 192 | 25 | 25/192 (13.0) | wound samples were collected by using a sterile cotton swab | Antimicrobial susceptibility -PCR | ([141](#_ENREF_141)) |
| Nickerson | nr | 2009 | Thailand | Asia | 81 | 42 | 42/81 (51.8) | wound,urine,tracheal aspirates,blood,cathather | disk diffusion | ([142](#_ENREF_142)) |
| Scangarella | nr | 2009 | Oman | Asia | 1975 | 134 | 134/1975 (6.7) | impetigo-bullous impetigo | disk diffusion | ([143](#_ENREF_143)) |
| Norazah | nr | 2003 | Malaysia | Asia | 79 | 9 | 9/79 (11.3) | dermatology patients and hospital inpatients | disk diffusion | ([144](#_ENREF_144)) |
| Norazah | nr | 2002 | Malaysia | Asia | 640 | 32 | 32/640 (5.0) | dermatology patients and hospital inpatients | disk diffusion | ([145](#_ENREF_145)) |
| Norazah | 1997-1999 | 2005 | Malaysia | Asia | 685 | 32 | 32/685 (4.6) | clincal apecimens | disk diffusion-agar dillution method | ([146](#_ENREF_146)) |
| Yıldız | nr | 2014 | Turkey | Europe | 397 | 32 | 32/397 (8.0) | blood, urine, wound, body fluids, and sputum | Antimicrobial Susceptibility Testing-DNA Extraction-PCR-disk agar diffusion test | ([147](#_ENREF_147)) |
| [Grohs](https://www.ncbi.nlm.nih.gov/pubmed/?term=Grohs%20P%5BAuthor%5D&cauthor=true&cauthor_uid=12499229) | nr | 2003 | France | Europe | 10 | 2 | 2/10 (20.0) | wound specimens | Antimicrobial susceptibility testing-PCR | ([148](#_ENREF_148)) |
| Arkwright | nr | 2002 | England | Europe | 194 | 74 | 74/194 (38.1) | Ocular isolates | agar dilution method | ([149](#_ENREF_149)) |
| Hoeger | nr | 2004 | Germany | Europe | 100 | 6 | 6/100 (6.0) | pus,blood culture, articular puncture,venous catheter | the phoenix Automated Microbiology system- disk diffusion | ([150](#_ENREF_150)) |
| Pfaller | 1997-2006 | 2010 | Canada | America | 217 | 14 | 14/217 (6.4) | nr | nr | ([151](#_ENREF_151)) |
| Pichon | 2011 | 2012 | UK | Europe | 165 | 4 | 4/165 (2.4) | skin surface, nostrils, upper throat, scars or pimples | Antibiotic susceptibility-PCR | ([152](#_ENREF_152)) |
| [Saginur](https://www.researchgate.net/profile/Raphael_Saginur) | nr | 2006 | Canada | America | 23 | 3 | 3/23 (13.0) | recovered from storage | disk diffusion | ([153](#_ENREF_153)) |
| Rennie | 1995-2005 | 2006 | Canada | America | 2302 | 65 | 65/2302 (2.8) | nr | nr | ([154](#_ENREF_154)) |
| Rijnders | nr | 2010 | Netherland | Europe | 129 | 9 | 9/129 (6.9) | blood | disk diffusion method | ([155](#_ENREF_155)) |
| Flamm | 2004-2013 | 2016 | USA | America | 48 | 11 | 11/48 (22.9) | nr | nr | ([156](#_ENREF_156)) |
| jones | 2008 to 2009 | 2011 | US | America | 7339 | 26 | 26/7339 (0.3) | Nasal, throat and axillary swabs | Antibiotic disk susceptibility testing | ([157](#_ENREF_157)) |
| Rohani | nr | 2000 | Malaysia | Asia | 390 | 14 | 14/390 (3.5) | nr | nr | ([158](#_ENREF_158)) |
| Rørtveit | 2003 | 2003 | Norway | Europe | 79 | 67 | 67/79 (84.8) | skin, wound swabs, pus, blood, tracheal aspirates, sputum, urine and tissue | E-test | ([159](#_ENREF_159)) |
| [Klein](https://www.sciencedirect.com/science/article/abs/pii/S0924857918303248?via%3Dihub#!) | 2012-2016 | 2019 | Germany | Europe | 2475 | 297 | 297/2475 (12.0) | nr | MIC | ([160](#_ENREF_160)) |
| Lemaire | 2008 | 2011 | US | America | 94 | 4 | 4/94 (4.25) | nr | nr | ([161](#_ENREF_161)) |
| Koning | 1987-2001 | 2006 | Netherland | Europe | 357 | 64 | 64/357 (17.9) | skin swab | nr | ([162](#_ENREF_162)) |
| Mehdi | 2007-2011 | 2016 | Pakistan | Asia | 17 | 9 | 9/17 (52.9) | Skin swabs | Antimicrobial susceptibility | ([163](#_ENREF_163)) |
| Saleem | nr | 2017 | Pakistan | Asia | 234 | 58 | 58/234 (24.7) | nr | microdilution | ([164](#_ENREF_164)) |
| Salmanov | nr | 2019 | Ukraine | Europe | 516 | 15 | 15/516 (2.9) | nr | nr | ([165](#_ENREF_165)) |
| Boswihi | 2016 | 2018 | Kuwait | Asia | 1327 | 89 | 89/1327 (6.7) | nr | agar dilution | ([166](#_ENREF_166)) |
| Perwaiz | 2004-2005 | 2007 | Pakistan | Asia | 190 | 2 | 2/190 (1.0) | nr | nr | ([167](#_ENREF_167)) |
| Samra | nr | 2005 | Israel | Europe | 150 | 6 | 6/150 (4.0) | from routine cultures | disk diffusion | ([168](#_ENREF_168)) |
| SǍndulescu | nr | 2014 | Romania | Europe | 149 | 7 | 7/149 (4.6) | kin and soft tissue | MIC / MBC | ([169](#_ENREF_169)) |
| [Park](https://www.ncbi.nlm.nih.gov/pubmed/?term=Park%20SH%5BAuthor%5D&cauthor=true&cauthor_uid=26512169) | nr | 2015 | Korea | Asia | 497 | 18 | 18/497 (3.6) | bloodstream, respiratory tract infections, and skin | Broth microdilution | ([170](#_ENREF_170)) |
| Sasirekha | 2010-2011 | 2012 | India | Asia | 153 | 102 | 102/153 (66.6) | blood, urine, pus, wound, respiratory tract, cerebrospinal fluid | disk diffusion | ([171](#_ENREF_171)) |
| Scerri | nr | 2013 | Malta | Europe | 329 | 23 | 23/329 (6.9) | nr | nr | ([172](#_ENREF_172)) |
| Senok | nr | 2018 | Saudi Arabia | Asia | 29 | 4 | 4/29 (13.7) | nr | nr | ([173](#_ENREF_173)) |
| Nishijima | 1999-2000 | 2002 | Japan | Asia | 229 | 11 | 11/229 (4.8) | persistent bacteremia and endocarditis | nr | ([174](#_ENREF_174)) |
| Seydi | 1996-2002 | 2004 | Senegal | Africa | 130 | 4 | 4/130 (3.0) | nr | disk diffusion | ([175](#_ENREF_175)) |
| Sfeir | nr | 2014 | Lebanon | Asia | 133 | 11 | 11/133 (8.2) | pediatric burn patients | disk diffusion | ([176](#_ENREF_176)) |
| Akcali | nr | 2001 | Turkey | Europe | 105 | 3 | 3/105 (2.8) | clinical isolates | VITEK 2 | ([177](#_ENREF_177)) |
| Souli | 2012-2013 | 2016 | Greece | Europe | 980 | 330 | 330/980 (33.6) | nr | Kirby - Bauer antibiotic testing | ([178](#_ENREF_178)) |
| Stevens | 2005-2006 | 2006 | South Australia | Oceania | 524 | 7 | 7/524 (1.3) | nr | disk diffusion | ([179](#_ENREF_179)) |
| Strandén | 2006-2010 | 2012 | Swiss | Europe | 258 | 14 | 14/258 (5.4) | clinical specimens | Antibiotic susceptibility testing | ([180](#_ENREF_180)) |
| Sule | 2002 | 2007 | Denmark | Europe | 62 | 31 | 31/62 (50.0) | hospitalized patients | MIC | ([181](#_ENREF_181)) |
| Rørtveit | 2001-2009 | 2011 | Norway | Europe | 278 | 197 | 197/278 (70.8) | children aged 0-12 years | disk diffusion | ([182](#_ENREF_182)) |
| Rørtveit | 2001-2012 | 2014 | Norway | Europe | 296 | 198 | 198/296 (66.8) | cutaneous wound infections,blood cultures, sputum samples and other infection sites | MALDI-TOF or VITEK | ([183](#_ENREF_183)) |
| Swathi | 2016 | 2019 | India | Asia | 63 | 13 | 13/63 (20.6) | nr | agar dilution / PFGE | ([184](#_ENREF_184)) |
| Titov | 2008-2010 | 2011 | Belarus | Europe | 943 | 38 | 38/943 (4.0) | clinical samples | E. test and MIC | ([185](#_ENREF_185)) |
| [Treesirichod](https://www.ncbi.nlm.nih.gov/pubmed/?term=Treesirichod%20A%5BAuthor%5D&cauthor=true&cauthor_uid=23668464) | nr | 2012 | Thailand | Asia | 128 | 4 | 4/128 (3.1) | Nasal | microarray, Vitek 2 | ([186](#_ENREF_186)) |
| Ertem | nr | 2013 | Turkey | Europe | 60 | 5 | 5/60 (8.3) | nr | nr | ([187](#_ENREF_187)) |
| Tveten | 1994-98 | 2003 | Norway | Europe | 87 | 30 | 30/87 (34.4) | various skin infections | agar dilution-MIC | ([188](#_ENREF_188)) |
| Udo | 2016-2017 | 2019 | Kuwait | Asia | 3801 | 1998 | 1998/3801 (52.5) | blood | disk diffusion method | ([189](#_ENREF_189)) |
| Udo | 2011 | 2014 | Oman | Asia | 79 | 12 | 12/79 (15.1) | nasal and pharyngea | disk diffusion | ([190](#_ENREF_190)) |
| Udo | nr | 2008 | Kuwait | Asia | 1846 | 557 | 557/1846 (30.1) | wound,urine,sputum,blood,cathather | nr | ([191](#_ENREF_191)) |
| Uluǧ | nr | 2009 | Turkey | Europe | 41 | 4 | 4/41 (9.7) | blood | Vitek2,E. test | ([192](#_ENREF_192)) |
| Vallières | 2000-2014 | 2015 | UK | Europe | 36 | 11 | 11/36 (30.5) | nr | nr | ([193](#_ENREF_193)) |
| [Gostev](https://www.researchgate.net/profile/Vladimir_Gostev) | nr | 2017 | Russia | Europe | 518 | 10 | 10/518 (1.9) | hospitalized patient | PCR | ([194](#_ENREF_194)) |
| Vourli | nr | 2005 | Greece | Europe | 20 | 18 | 18/20 (90.0) | eczema lesion | disk method | ([195](#_ENREF_195)) |
| [Alfouzan](https://www.nature.com/articles/s41598-019-54794-8#auth-1) | 2016 | 2019 | Kuwait | Asia | 209 | 133 | 133/209 (63.6) | skin swabs | E. test | ([196](#_ENREF_196)) |
| Wasserman | nr | 2014 | South Africa | Africa | 996 | 150 | 150/996 (15.0) | skin | disk diffusion tests,PFGE | ([197](#_ENREF_197)) |
| Wang | 2006-2010 | 2015 | Taiwan | Asia | 670 | 97 | 97/670 (14.4) | swab of skin | KirbyBauer’s disk diffusion method | ([198](#_ENREF_198)) |
| Wiśniewska | 1990-1998 | 2000 | Poland | Europe | 225 | 35 | 35/225 (15.5) | clinical samples | nr | ([199](#_ENREF_199)) |
| Wisniewska | 1997-2000 | 2002 | Poland | Europe | 190 | 6 | 6/190 (3.1) | nasal | disk diffusion | ([200](#_ENREF_200)) |
| Liu | nr | 2017 | China | Asia | 34 | 1 | 1/34 (2.9) | nr | MIC | ([201](#_ENREF_201)) |
| [Chen](https://www.researchgate.net/profile/Xu_Chen151) | 2009 | 2012 | China | Asia | 85 | 1 | 1/85 (1.1) | nr | nr | ([202](#_ENREF_202)) |
| Huang | nr | 2013 | Taiwan | Asia | 116 | 4 | 4/116 (3.4) | different clinical samples | microarray | ([97](#_ENREF_97)) |
| Yilmaz | nr | 2017 | Turkey | Europe | 97 | 12 | 12/97 (12.3) | clinical samples | disk diffusion | ([203](#_ENREF_203)) |
| Liu | nr | 2016 | China | Asia | 1946 | 199 | 199/1946 (10.2) | blood, wound | disk diffusion ,E. test | ([204](#_ENREF_204)) |
| Baek | 2010–2013 | 2016 | South Korea | Asia | 965 | 425 | 425/965 (44.0) | bone tissue and sinus-tract | Disk diffusion | ([205](#_ENREF_205)) |
| Huang | 2009-2011 | 2018 | Taiwan | Asia | 30 | 6 | 6/30 (20.0) | respiratory samples | nr | ([206](#_ENREF_206)) |
| Rashid | nr | 2012 | Pakistan | Asia | 49 | 12 | 12/49 (24.4) | hospital patients, healthy medical personnels, and healthy carriers | nr | ([207](#_ENREF_207)) |
| Zinn | 1996 | 2004 | Belgium | Europe | 100 | 1 | 1/100 (1.0) | skin and soft tissue infections | PFGE | ([208](#_ENREF_208)) |
| Zinn | 1996 | 2004 | Argentina | Europe | 102 | 1 | 1/102 (0.9) | wound,blood,pus,sputum, respiratory samples | disk diffusion | ([208](#_ENREF_208)) |
| Zinn | 1996 | 2004 | Finland | Europe | 110 | 1 | 1/110 (0.9) | respiratory specimen, swab, pus, tissue, and blood and cerebrospinal | disk diffusion | ([208](#_ENREF_208)) |
| Zinn | 1996 | 2004 | Malaysia | Asia | 221 | 1 | 1/221 (0.4) | blood | VITEK 2 system | ([208](#_ENREF_208)) |
| Zinn | 1996 | 2004 | Germany | Europe | 145 | 3 | 3/154 (2.0) | clinical strains | MIC | ([208](#_ENREF_208)) |
| Zinn | 1996 | 2004 | Sweden | Europe | 200 | 3 | 3/200 (1.5) | clinical samples | disk-diffusion | ([208](#_ENREF_208)) |
| Zinn | 1996 | 2004 | Spain | Europe | 100 | 4 | 4/100 (4.0) | nr | MIC | ([208](#_ENREF_208)) |
| Zinn | 1996 | 2004 | Norway | Europe | 200 | 4 | 4/200 (2.0) | burn centers | disk diffusion | ([208](#_ENREF_208)) |
| Zinn | 1996 | 2004 | Denmark | Europe | 100 | 5 | 5/100 (5.0) | Nasal | PFGE, MLST, disk diffusion | ([208](#_ENREF_208)) |
| Zinn | 1996 | 2004 | Western Australia | Oceania | 100 | 5 | 5/100 (5.0) | wound swabs, urine ,blood cultures, sputum and other samples | Antimicrobial susceptibility testing | ([208](#_ENREF_208)) |
| Zinn | 1996 | 2004 | England | Europe | 200 | 6 | 6/200 (3.0) | infection sites | agar dilution method | ([208](#_ENREF_208)) |
| Zinn | 1996 | 2004 | New Zealand | Oceania | 200 | 13 | 13/200 (6.5) | skin infections | Antimicrobial susceptibility testing | ([208](#_ENREF_208)) |
| Zinn | 1996 | 2004 | Kuwait | Asia | 100 | 20 | 20/100 (20.0) | Nasal swabs | Antimicrobial Susceptibility Testing-PFGE-PCR | ([208](#_ENREF_208)) |
| Zinn | 1996 | 2004 | Greece | Europe | 100 | 49 | 49/100 (49.0) | Nasal | Kirby Bauer disk diffusion | ([208](#_ENREF_208)) |

MIC; Minimal Inhibitory Concentration, MBC; minimum bactericidal concentration, PCR; Polymerase Chain Reaction, PFGE; Pulsed-field gel electrophoresis, MLST; Multilocus sequence typing, nr; not report

Table S2. Characteristics of included studies which studied FRMSSA.

| **First author** | **Time of study** | **Published time** | **Country** | **Continent** | **S.**  **aureus** | **MSSA** | **MSSA/S. aureus** | **FR-MSSA** | **FRMSSA/S. aureus** | **FRMSSA/MSSA** | **Source of samples** | **Methods** | **Ref** |
| --- | --- | --- | --- | --- | --- | --- | --- | --- | --- | --- | --- | --- | --- |
| Sarkar | 2013-2014 | 2016 | Saudi Arabia | Asia | 32 | 31 | 31/32 (96.8) | 4 | 4/32 (12.5) | 4/31 (12.9) | nasal swab sample | Antimicrobial susceptibility testing-DNA microarray | ([5](#_ENREF_5)) |
| [Senok](https://www.liebertpub.com/doi/10.1089/mdr.2019.0318) | nr | 2019 | UAE | Asia | 29 | 23 | 23/29 (79.3) | 2 | 2/29 (6.8) | 2/23 (8.6) | Nasal swabs | nr | ([6](#_ENREF_6)) |
| [Al-Sweih](https://www.ncbi.nlm.nih.gov/pubmed/?term=Al%20Sweih%20N%5BAuthor%5D&cauthor=true&cauthor_uid=16433190) | nr | 2005 | Kuwait | Asia | 1983 | 1328 | 1328/1983 (66.9) | 319 | 319/1983 (16.0) | 319/1328 (24.0) | different sources | E. test, disk diffusion and Vitek | ([12](#_ENREF_12)) |
| Aldasouqi | 2015 | 2019 | Jordan | Asia | 113 | 53 | 53/113 (46.9) | 12 | 12/113 (10.6) | 12/53 (22.6) | urine,blood,tissue,respiratory secretion | disk diffusion | ([14](#_ENREF_14)) |
| Somily | 2005-2006 | 2012 | Saudi Arabia | Asia | 122 | 76 | 76/122 (62.2) | 12 | 12/122 (9.8) | 12/76 (15.7) | soft tissue including pus, joint fluid, and blood | disk diffusion,E. test | ([15](#_ENREF_15)) |
| Hasani | nr | 2013 | Iran | Asia | 150 | 69 | 69/150 (46.0) | 3 | 3/150 (2.0) | 3/69 (4.3) | blood,urine, postoperative wound, synovial fluid, sputum | E-test/PCR | ([16](#_ENREF_16)) |
| Al-Talib | 2008 | 2015 | Malaysia | Asia | 158 | 124 | 124/158 (78.4) | 20 | 20/158 (12.6) | 20/124 (16.1) | blood, cerebrospinal fluid, respiratory tract, eye, pus and wound, urine, genital specimens, environment and other sites | Antibiotic susceptibility testing | ([17](#_ENREF_17)) |
| Doudoulakakis | 2013-2016 | 2017 | Greece | Europe | 102 | 99 | 99/102 (97.0) | 95 | 95/102 (93.1) | 95/99 (95.9) | skin | PCR-molecular typing | ([21](#_ENREF_21)) |
| Atmaca | nr | 2001 | Turkey | Europe | 103 | 48 | 48/103 (46.6) | 2 | 2/103 (1.9) | 2/48 (4.1) | nr | disk diffusion | ([23](#_ENREF_23)) |
| Brosnikoff | nr | 2009 | Canada | America | 602 | 502 | 502/602 (83.3) | 38 | 38/602 (6.3) | 38/502 (7.5) | clinical strains | disk diffusion | ([31](#_ENREF_31)) |
| Chen | 2002-2007 | 2010- | Taiwan | Asia | 71 | 26 | 26/71 (36.6) | 26 | 26/71 (36.6) | 26/26 (100.0) | Pus,Blood ,Urine ,Sputum,Miscellaneous | broth microdilution/PCR | ([40](#_ENREF_40)) |
| Demir | 2007-2008 | 2012 | Turkey | Europe | 242 | 165 | 165/242 (68.1) | 2 | 2/242 (0.8) | 2/165 (1.2) | wound | Kirby–Bauer disk diffusion | ([51](#_ENREF_51)) |
| Brown | 1998 | 2002 | UK | Europe | 2542 | 1934 | 1934/2542 (76.0) | 116 | 116/2542 (4.5) | 116/1934 (5.9) | lesional skin,nonlesional skin,anterior nares | Disk diffusion | ([73](#_ENREF_73)) |
| Brown | 2000 | 2002 | UK | Europe | 2893 | 1886 | 1886/2893 (65.1) | 156 | 156/2893 (5.3) | 156/1886 (8.2) | nasal | disk diffusion | ([73](#_ENREF_73)) |
| Brown | 1999 | 2002 | UK | Europe | 2883 | 1994 | 1994/2883 (69.1) | 177 | 177/2883 (6.1) | 177/1994 (8.8) | Clinical samples | nr | ([73](#_ENREF_73)) |
| Brown | 2001 | 2002 | UK | Europe | 2947 | 1610 | 1610/2947 (54.6) | 185 | 185/2947 (6.2) | 185/1610 (11.4) | nr | hybridization | ([73](#_ENREF_73)) |
| Fangyou | 2012-2013 | 2015 | China | Asia | 392 | 211 | 211/392 (53.8) | 7 | 7/392 (1.7) | 7/211 (3.3) | blood samples | nr | ([80](#_ENREF_80)) |
| [Randrianirina](https://www.ncbi.nlm.nih.gov/pubmed/?term=Randrianirina%20F%5BAuthor%5D&cauthor=true&cauthor_uid=17521424) | 2001-2005 | 2007 | Madagascar | Africa | 529 | 493 | 493/529 (93.1) | 7 | 7/529 (1.3) | 7/493 (1.4) | sputum, pus , catheter, blood and exudates | disk diffusion-MIC | ([85](#_ENREF_85)) |
| Coombs | 2011 | 2013 | Australia | Oceania | 2357 | 1644 | 1644/2357 (69.7) | 55 | 55/2357 (2.3) | 55/1644 (3.3) | Pus,Blood ,Urine ,Sputum,Miscellaneous | broth microdilution/PCR | ([88](#_ENREF_88)) |
| Huang | nr | 2013 | China | Asia | 116 | 97 | 97/116 (83.6) | 3 | 3/116 (2.5) | 3/97 (3.0) | nosocomial | disk diffusion method | ([97](#_ENREF_97)) |
| Balawi | 2014 | 2017 | Arabia | Asia | 54 | 28 | 28/54 (51.8) | 14 | 14/54 (25.9) | 14/28 (50.0) | nosocomial | disk diffusion method | ([98](#_ENREF_98)) |
| Błażewicz | 2014-2015 | 2017 | Poland | Europe | 148 | 137 | 137/148 (92.5) | 28 | 28/148 (18.9) | 28/137 (20.4) | nasal | MIC | ([99](#_ENREF_99)) |
| Jappe | 2003-2005 | 2008 | Germany | Europe | 130 | 90 | 90/130 (69.2) | 6 | 6/130 (4.6) | 6/90 (6.6) | wound sepsis samples wound infections | Antibiotic Susceptibility Testing | ([102](#_ENREF_102)) |
| Klein | 2009–2014 | 2016 | Germany | Europe | 526 | 448 | 448/526 (85.1) | 13 | 13/526 (2.4) | 13/448 (2.9) | deep throat swab | E. test | ([113](#_ENREF_113)) |
| Livermore | 1990 | 2002 | UK | Europe | 4800 | 4718 | 4718/4800 (98.2) | 1 | 1/4800 (0.0) | 1/4718 (0.0) | deep throat swab" | E. test | ([117](#_ENREF_117)) |
| Livermore | 1991 | 2002 | UK | Europe | 4966 | 4892 | 4892/4966 (98.5) | 132 | 132/4966 (2.6) | 132/4892 (2.6) | sputum samples | nr | ([117](#_ENREF_117)) |
| Livermore | 1992 | 2002 | UK | Europe | 5049 | 4918 | 4918/5049 (97.4) | 101 | 101/5049 (2.0) | 101/4918 (2.0) | deep throat swab" | E. test | ([117](#_ENREF_117)) |
| Livermore | 1993 | 2002 | UK | Europe | 5741 | 5535 | 5535/5741 (96.4) | 127 | 127/5741 (2.2) | 127/5535 (2.2) | sputum samples | nr | ([117](#_ENREF_117)) |
| Livermore | 1994 | 2002 | UK | Europe | 5960 | 5477 | 5477/5960 (91.8) | 112 | 112/5960 (1.8) | 112/5477 (2.0) | deep throat swab | E. test | ([117](#_ENREF_117)) |
| Livermore | 1995 | 2002 | UK | Europe | 6588 | 5718 | 5718/6588 (86.7) | 142 | 142/6588 (2.1) | 142/5718 (2.4) | sputum samples | nr | ([117](#_ENREF_117)) |
| Livermore | 1996 | 2002 | UK | Europe | 7779 | 6138 | 6138/7779 (78.9) | 208 | 208/7779 (2.6) | 208/6138 (3.3) | deep throat swab | antimicrobial susceptibility tests | ([117](#_ENREF_117)) |
| Livermore | 1997 | 2002 | UK | Europe | 9328 | 6372 | 6372/9328 (68.3) | 230 | 230/9328 (2.4) | 230/6327 (3.6 | dermatology inpatients | nr | ([117](#_ENREF_117)) |
| Livermore | 1998 | 2002 | UK | Europe | 9374 | 6187 | 6187/9374 (66.0) | 266 | 266/9374 (2.8) | 266/6187 (4.2) | Eschar Muscle Blood Lung | agar dilution test and E. test | ([117](#_ENREF_117)) |
| Livermore | 1999 | 2002 | UK | Europe | 10813 | 6845 | 6845/10813 (63.3) | 284 | 284/10813 (2.6) | 284/6845 (4.1) | joint fluids /Bone and joint infections | PCR | ([117](#_ENREF_117)) |
| Livermore | 2000 | 2002 | UK | Europe | 11635 | 6749 | 6749/11635 (58.0) | 459 | 459/11635 (3.9) | 459/6749 (6.8) | skin & blood | Kirby-Bauer disk diffusion | ([117](#_ENREF_117)) |
| Livermore | 2001 | 2002 | UK | Europe | 12631 | 7200 | 7200/12631 (57.0) | 541 | 541/12631 (4.2) | 541/7200 (7.5) | Skin infection | disk diffusion | ([117](#_ENREF_117)) |
| Groome | 2005-2006 | 2012 | South Africa | Africa | 161 | 98 | 98/161 (60.8) | 5 | 5/161 (3.1) | 5/98 (5.1) | blood | nr | ([124](#_ENREF_124)) |
| Dinić | nr | 2013 | Serbia | Asia | 1381 | 1258 | 1258/1381 (91.0) | 119 | 119/1381 (8.6) | 119/1258 (9.4) | blood | nr | ([127](#_ENREF_127)) |
| Morrissey | nr | 2004 | UK | Europe | 158 | 123 | 123/158 (77.8) | 3 | 3/158 (1.8) | 3/123 (2.4) | skin swabs | microdilution breakpoint method (VITEK 2 ) or the E. test | ([137](#_ENREF_137)) |
| Sohail | 2012-2016 | 2018 | Pakistan | Asia | 344 | 141 | 141/344 (40.9) | 29 | 29/344 (8.4) | 29/141 (20.5) | skin swabs | disk diffusion | ([139](#_ENREF_139)) |
| Nergiz | 2001-2011 | 2012 | Turkey | Europe | 192 | 101 | 101/192 (52.6) | 5 | 5/192 (2.6) | 5/101 (4.9) | wound samples were collected by using a sterile cotton swab | biochemical and molecular methods-Antimicrobial susceptibility of isolates-PCR- spa typing | ([141](#_ENREF_141)) |
| Nickerson | nr | 2009 | Thailand | Asia | 81 | 58 | 58/81 (71.6) | 39 | 39/81 (48.1) | 39/58 (67.2) | wound,urine,tracheal aspirates,blood,cathather | disk diffusion | ([142](#_ENREF_142)) |
| Norazah | nr | 2003 | Malaysia | Asia | 79 | 79 | 79/79 (100.0) | 9 | 9/79 (11.3) | 9/79 (11.3) | dermatology patients and hospital inpatients | disk diffusion | ([144](#_ENREF_144)) |
| Pfaller | 1997-2006 | 2010 | Canada | America | 217 | 116 | 116/217 (53.4) | 7 | 7/217 (3.2) | 7/116 (6.0) | nr | nr | ([151](#_ENREF_151)) |
| Rennie | 1995-2005 | 2006 | Canada | America | 2302 | 2062 | 2062/2302 (89.5) | 55 | 55/2302 (2.3) | 55/2062 (2.6) | nr | nr | ([154](#_ENREF_154)) |
| Rohani | nr | 2000 | Malaysia | Asia | 390 | 235 | 235/390 (60.2) | 9 | 9/390 (2.3) | 9/235 (3.8) | nr | nr | ([158](#_ENREF_158)) |
| Sasirekha | 2010-2011 | 2012 | India | Asia | 153 | 111 | 111/153 (72.5) | 74 | 74/153 (48.3) | 74/111 (66.6) | blood,urine,pus,wound,respiratory trct,cerebrospinal fluid | disk diffusion | ([171](#_ENREF_171)) |
| Senok | nr | 2018 | Saudi Arabia | Asia | 29 | 23 | 23/29 (79.3) | 2 | 2/29 (6.8) | 2/23 (8.6) | nr | nr | ([173](#_ENREF_173)) |
| Seydi | 1996-2002 | 2004 | Senegal | Africa | 130 | 56 | 56/130 (43.0) | 1 | 1/130 (0.7) | 1/56 (1.7) | nr | disk diffusion | ([175](#_ENREF_175)) |
| Akcali | nr | 2001 | Turkey | Europe | 105 | 68 | 68/105 (64.7) | 1 | 1/105 (0.9) | 1/68 (1.4) | clinical isolates | minimum inhibitory concentrations using VITEK 2 system | ([177](#_ENREF_177)) |
| Souli | 2012-2013 | 2016 | Greece | Europe | 980 | 608 | 608/980 (62.0) | 106 | 106/980 (10.8) | 106/608 (17.4) | nr | Antimicrobial susceptibility -Kirby - Bauer antibiotic testing- | ([178](#_ENREF_178)) |
| Sule | 2002 | 2007 | Denmark | Europe | 62 | 60 | 60/62 (96.7) | 31 | 31/62 (50.0) | 31/60 (51.6) | hospitalized patients. | The minimal inhibitory concentration | ([181](#_ENREF_181)) |
| Swathi | 2016 | 2019 | India | Asia | 63 | 62 | 62/63 (98.4) | 13 | 13/63 (20.6) | 13/62 (20.9) | nr | agar dilution / PFGE | ([184](#_ENREF_184)) |
| [Treesirichod](https://www.ncbi.nlm.nih.gov/pubmed/?term=Treesirichod%20A%5BAuthor%5D&cauthor=true&cauthor_uid=23668464) | nr | 2012 | Thailand | Asia | 128 | 128 | 128/128 (100.0) | 4 | 4/128 (3.1) | 4/128 (3.1) | Nasal | microarray,Vitek 2 | ([186](#_ENREF_186)) |
| Huang | nr | 2013 | Taiwan | Asia | 116 | 97 | 97/116 (83.6) | 3 | 3/116 (2.5) | 3/97 (3.0) | different clinical samples | microarray | ([97](#_ENREF_97)) |
| Liu | nr | 2016 | China | Asia | 1946 | 1895 | 1895/1946 (97.3) | 27 | 27/1946 (1.3) | 27/1895 (1.4) | blood, wound | disk diffusion ,E. test | ([204](#_ENREF_204)) |
| Baek | 2010–2013 | 2016 | South Korea | Asia | 965 | 508 | 508/965 (52.6) | 202 | 202/965 (20.9) | 202/508 (39.7) | bone tissue and sinus-tract | Disk diffusion | ([205](#_ENREF_205)) |
| Huang | 2009-2011 | 2018 | Taiwan | Asia | 30 | 23 | 23/30 (76.6) | 2 | 2/30 (6.6) | 2/23 (8.6) | respiratory samples | nr | ([206](#_ENREF_206)) |
| Zinn | 1996 | 2004 | Belgium | Europe | 100 | 70 | 70/100 (70.0) | 1 | 1/100 (1.0) | 1/7 (1.4) | skin and soft tissue infections | PFGE | ([208](#_ENREF_208)) |
| Zinn | 1996 | 2004 | Finland | Europe | 110 | 108 | 108/110 (98.1) | 1 | 1/110 (0.9) | 1/108 (0.9) | respiratory specimen, swab, pus, tissue, and blood and cerebrospinal | disk diffusion | ([208](#_ENREF_208)) |
| Zinn | 1996 | 2004 | Malaysia | Asia | 221 | 113 | 113/221 (51.1) | 1 | 1/221 (0.4) | 1/113 (0.8) | blood | VITEK 2 sysytem | ([208](#_ENREF_208)) |
| Zinn | 1996 | 2004 | Germany | Europe | 145 | 136 | 136/145 (93.7) | 3 | 3/145 (2.0) | 3/136 (2.2) | clinical strains | disk-diffusion, agar dilution | ([208](#_ENREF_208)) |
| Zinn | 1996 | 2004 | Sweden | Europe | 200 | 198 | 198/200 (99.0) | 3 | 3/200 (1.5) | 3/198 (1.5) | clinical samples | disk-diffusion | ([208](#_ENREF_208)) |
| Zinn | 1996 | 2004 | Spain | Europe | 100 | 72 | 72/100 (72.0) | 4 | 4/200 (4.0) | 4/72 (5.5) | nr | MIC | ([208](#_ENREF_208)) |
| Zinn | 1996 | 2004 | Norway | Europe | 200 | 200 | 200/200 (100.0) | 4 | 4/200 (2.0) | 4/200 (2.0) | four burn centers | disk diffusion | ([208](#_ENREF_208)) |
| Zinn | 1996 | 2004 | Denmark | Europe | 100 | 100 | 100/100 (100.0) | 5 | 5/100 (5.0) | 5/100 (5.0) | Nasal | PFGE, MLST, disk diffusion | ([208](#_ENREF_208)) |
| Zinn | 1996 | 2004 | Western Australia | Oceania | 100 | 90 | 90/100 (90.0) | 5 | 5/100 (5.0) | 5/90 (5.5) | wound swabs, urine, blood cultures, sputum and other samples | Antimicrobial susceptibility testing | ([208](#_ENREF_208)) |
| Zinn | 1996 | 2004 | England | Europe | 200 | 162 | 162/200 (81.0) | 4 | 4/200 (2.0) | 4/162 (2.4) | infection sites | agar dilution method | ([208](#_ENREF_208)) |
| Zinn | 1996 | 2004 | New Zealand | Oceania | 200 | 180 | 180/200 (90.0) | 11 | 11/200 (5.5) | 11/180 (6.1) | skin infections | Antimicrobial susceptibility testing | ([208](#_ENREF_208)) |
| Zinn | 1996 | 2004 | Kuwait | Asia | 100 | 69 | 69/100 (69.0) | 18 | 18/100 (18.0) | 18/69 (26.0) | Nasal swabs | Antimicrobial Susceptibility Testing-PFGE-PCR | ([208](#_ENREF_208)) |
| Zinn | 1996 | 2004 | Greece | Europe | 100 | 37 | 37/100 (37.0) | 33 | 33/100 (33.0) | 33/37 (89.1) | Nasal | Kirby Bauer disk diffusion | ([208](#_ENREF_208)) |

MIC; Minimal Inhibitory Concentration, MBC; minimum bactericidal concentration, PCR; Polymerase Chain Reaction, PFGE; Pulsed-field gel electrophoresis, MLST; Multilocus sequence typing, nr; not report

Table S3. Characteristics of included studies which studied FRMRSA.

| **First author** | **Time of study** | **Published time** | **Country** | **Continent** | **S.**  **aureus** | **MRSA** | **MRSA/S. aureus** | **FR-MRSA** | **FR-MRSA/MRSA** | **FRMRSA/S. aureus** | **Source of samples** | **Methods** | **Ref** |
| --- | --- | --- | --- | --- | --- | --- | --- | --- | --- | --- | --- | --- | --- |
| Akpabie | 2003 | 2004 | France | Europe | 213 | 143 | 143/213 (67.1) | 3 | 3/143 (2.09) | 3/213 (1.4) | nr | disk diffusion test | ([2](#_ENREF_2)) |
| Larsen | 1993 - 2004 | 2007 | Denmark | Europe | 294 | 294 | 294/294 (100.0) | 277 | 277/294 (94.2) | 277/294 (94.2) | nr | agar dilution | ([4](#_ENREF_4)) |
| [Senok](https://www.liebertpub.com/doi/10.1089/mdr.2019.0318) | nr | 2019 | UAE | Asia | 29 | 6 | 6/29 (20.6) | 2 | 2/6 (33.3) | 2/29 (6.8) | Nasal swabs | nr | ([6](#_ENREF_6)) |
| Senok | 2017 | 2019 | Saudi Arabia | Asia | 125 | 125 | 125/125 (100.0) | 54 | 54/125 (43.2) | 54/125 (43.2) | nr | antibiotic susceptibility testing-DNA microarray | ([7](#_ENREF_7)) |
| Ahmadi | 2017-2018 | 2019 | Iran | Asia | 181 | 55 | 55/181 (30.3) | 1 | 1/55 (1.81) | 1/181 (0.5) | anterior nares | Agar disk diffusion method | ([8](#_ENREF_8)) |
| [Al Sweih](https://www.ncbi.nlm.nih.gov/pubmed/?term=Al%20Sweih%20N%5BAuthor%5D&cauthor=true&cauthor_uid=16433190) | nr | 2005 | Kuwait | Asia | 1983 | 745 | 745/1983 (37.5) | 603 | 603/745 (80.9) | 603/1983 (30.4) | different sources | Etest, disk diffusion and Vitek | ([12](#_ENREF_12)) |
| [Abouelfetouh](https://www.researchgate.net/profile/Alaa_Abouelfetouh) | 2011 | 2016 | Egypt | Africa | 81 | 81 | 81/81 (100.0) | 26 | 26/81 (32.0) | 26/81 (32.0) | pus,bloodmsputum,urine,BA lavage | PCR | ([13](#_ENREF_13)) |
| Aldasouqi | 2015 | 2019 | Jordan | Asia | 113 | 60 | 60/113 (53.0) | 24 | 24/60 (40.0) | 24/113 (21.2) | urine,blood,tissue,respiratory secretion | disk diffusion | ([14](#_ENREF_14)) |
| Somily | 2005-2006 | 2012 | Saudi Arabia | Asia | 122 | 46 | 46/122 (37.7) | 37 | 37/46 (80.4) | 37/122 (30.32) | soft tissue including pus, joint fluid, and blood | disk diffusion, E. test | ([15](#_ENREF_15)) |
| Hasani | nr | 2013 | Iran | Asia | 150 | 81 | 81/150 (54.0) | 4 | 4/81 (4.9) | 4/150 (2.6) | blood,urine, postoperative wound, synovial fluid, sputum | E-test/PCR | ([16](#_ENREF_16)) |
| Aqel | 2011-2012 | 2015 | Jordan | Asia | 56 | 56 | 56/56 (100.0) | 3 | 3/56 (5.3) | 3/56 (5.3) | nasal swabs | PCR | ([18](#_ENREF_18)) |
| Cabrera | 2010 | 2016 | Canada | America | 3036 | 3036 | 3036/3036 (100.0) | 246 | 246/3036 (8.1) | 246/3036 (8.1) | bloodstream infections, skin/soft tissue, respiratory, surgical site, urine and other sites. | PFGE | ([19](#_ENREF_19)) |
| Doudoulakakis | 2013-2016 | 2017 | Greece | Europe | 102 | 3 | 3/102 (2.94) | 2 | 2/3 (66.6) | 2/102 (1.9) | skin | PCR-molecular typing | ([21](#_ENREF_21)) |
| Shore | 1971-2004 | 2012 | Germany | Europe | 175 | 175 | 175/175 (100.0) | 21 | 21/175 (12.0) | 21/175 (12.0) | nr | multiplex PCR-DNA microarray analysis-spa typing | ([22](#_ENREF_22)) |
| Atmaca | nr | 2001 | turkey | Europe | 103 | 53 | 53/103 (51.4) | 10 | 10/53 (18.8) | 10/103 (9.7) | nr | disk diffusion | ([23](#_ENREF_23)) |
| [Willey](https://www.researchgate.net/profile/Barbara_Willey) | 2007-2008 | 2009 | Canada | America | 290 | 50 | 50/290 (17.2) | 17 | 17/50 (34.0) | 17/290 (17.2) | swabs from nares and wounds | nr | ([209](#_ENREF_209)) |
| Ahmad | nr | 2014 | Pakistan | Asia | 54 | 54 | 54/54 (100.0) | 9 | 9/54 (16.6) | 9/54 (16.6) | pus samples | Susceptibility test for antimicrobial agen-PFGE method-PCR | ([25](#_ENREF_25)) |
| Bauer | 1999-2007 | 2009 | Austria | Europe | 2542 | 2542 | 2542/2542 (100.0) | 81 | 81/2542 (3.1) | 81/2542 (3.1) | skin,wound,pus,abscess,nasal,miscellaneous,endotracheal | Kirby-Bauer disk diffusion method on Mueller-Hinton agar | ([26](#_ENREF_26)) |
| Berktold | 2005-2010 | 2012 | Australia | Oceania | 650 | 650 | 650/650 (100.0) | 84 | 84/650 (12.9) | 84/650 (12.9) | nr | nr | ([27](#_ENREF_27)) |
| Bhattacharya | 2009-2012 | 2016 | India | Asia | 1049 | 267 | 267/1049 (25.4) | 20 | 20/267 (7.4) | 20/1049 (1.9) | surgical procedures | Kirby Bauer technique | ([210](#_ENREF_210)) |
| Boswihi | 1992-2010 | 2016 | Kuwait | Asia | 400 | 400 | 400/400 (100) | 214 | 214/400 (53.5) | 214/400 (53.5) | nr | antibiogram | ([30](#_ENREF_30)) |
| Brosnikoff | nr | 2009 | Canada | America | 602 | 100 | 100/602 (16.6) | 6 | 6/100 (6.0) | 6/602 (0.9) | clinical strains | disk diffusion | ([31](#_ENREF_31)) |
| [Budimir](https://europepmc.org/search?query=AUTH:%22Ana%20Budimir%22) | 2001-2007 | 2016 | Croatia | Europe | 46 | 46 | 46/46 (100.0) | 1 | 1/46 (2.1) | 1/46 (2.1) | BLOOD | PFGE | ([32](#_ENREF_32)) |
| Horner | nr | 2012 | England | Europe | 888 | 888 | 888/888 (100.0) | 118 | 118/888 (13.2) | 118/888 (13.2) | nasal | disk susceptibility testing method | ([34](#_ENREF_34)) |
| Chang | 2003-2007 | 2011 | Taiwan | Asia | 236 | 125 | 125/236 (52.9) | 1 | 1/125 (0.8) | 1/236 (0.4) | wound/pus | disk diffusion | ([211](#_ENREF_211)) |
| Chen | 2002-2007 | 2010- | Taiwan | Asia | 71 | 45 | 45/71 (63.3) | 45 | 45/45 (100.0) | 45/71 (63.3) | Pus,Blood ,Urine ,Sputum,Miscellaneous | broth microdilution/PCR | ([40](#_ENREF_40)) |
| Chen | 2007 - 2008 | 2011 | Taiwan | Asia | 34 | 34 | 34/34 (100.0) | 25 | 25/34 (73.5) | 25/34 (73.5) | Pus,Blood ,Urine ,Sputum,Miscellaneous | broth microdilution/PCR | ([41](#_ENREF_41)) |
| Chuamuangphan | 2012 | 2013 | Korea | Asia | 156 | 52 | 52/156 (33.3) | 1 | 1/52 (1.92) | 1/156 (0.6) | Blood | disk diffusion | ([42](#_ENREF_42)) |
| Cirkovic | nr | 2010 | Serbia | Asia | 21 | 21 | 21/21 (100.0) | 2 | 2/21 (9.5) | 2/21 (9.5) | skin and soft tissue | MIC | ([43](#_ENREF_43)) |
| Decousser | 2011-2012 | 2015 | France | Europe | 367 | 367 | 367/367 (100.0) | 50 | 50/367 (13.6) | 50/367 (13.6) | from bacteraemia and osteoarticular infections | broth microdilution | ([50](#_ENREF_50)) |
| Demir | 2007-2008 | 2012 | Turkey | Europe | 242 | 77 | 77/242 (31.8) | 4 | 4/77 (5.1) | 4/242 (1.6) | wound | Kirby–Bauer disk diffusion | ([51](#_ENREF_51)) |
| Denis | 2002-2004 | 2005 | Belgian | Europe | 41 | 41 | 41/41 (100.0) | 23 | 23/41 (56.0) | 23/41 (56.0) | skin or soft tissue specimens from upper limbs, trunk , lower limbs, throat swab, blood and peritoneal fluid | disk diffusion | ([53](#_ENREF_53)) |
| Ghaith | 2019 | 2019 | Egypt | Africa | 250 | 250 | 250/250 (100.0) | 50 | 50/250 (20.0) | 50/250 (20.0) | wound and skin infections | PCR | ([54](#_ENREF_54)) |
| [Anastasiou](https://www.ijidonline.com/article/S1201-9712(11)60068-4/fulltext) | 2008-2010 | 2011 | Greece | Europe | 140 | 66 | 66/140 (47.1) | 3 | 3/66 (4.5) | 3/140 (2.1) | pus,blood,urine,trauma | agar dilution | ([55](#_ENREF_55)) |
| Udo | 2018 | 2020 | Kuwait | Asia | 97 | 97 | 97/97 (100.0) | 97 | 97/97 (100.0) | 97/97 (100.0) | nr | MIC / PCR | ([58](#_ENREF_58)) |
| Champion | 2008-2010 | 2013 | USA | America | 277 | 277 | 277/277 (100.0) | 8 | 8/277 (2.8) | 8/277 (2.8) | respiratory secretions | Antibiotic Susceptibility Testing, multiplex PCR-E-tests | ([59](#_ENREF_59)) |
| Udo | 1996-2001 | 2006 | Kuwait | Asia | 88 | 88 | 88/88 (100.0) | 74 | 74/88 (84.0) | 74/88 (84.0) | skin swabs | disk diffusion | ([60](#_ENREF_60)) |
| Udo | nr | 2010 | Kuwait | Asia | 135 | 135 | 135/135 (100.0) | 57 | 57/135 (42.2) | 57/135 (42.2) | wound swabs, | nr | ([61](#_ENREF_61)) |
| Udo | 1994-2004 | 2006 | Kuwait | Asia | 5644 | 5644 | 5644/5644 (100.0) | 756 | 756/5644 (13.3) | 756/5644 (13.3) | abscesses, nasal swabs, ear swabs | nr | ([62](#_ENREF_62)) |
| Udo | 2011–2015 | 2017 | Kuwait | Asia | 6922 | 6922 | 6922/6922 (100.0) | 2858 | 2858/6922 (41.2) | 2858/6922 (41.2) | swabs, tracheal aspirates, high vaginal swabs , sputum and eye swab | nr | ([65](#_ENREF_65)) |
| Udo | nr | 2008 | Bahrain | Asia | 53 | 53 | 53/53 (100.0) | 49 | 49/53 (92.4) | 49/53 (92.4) | nr | disk diffusion | ([67](#_ENREF_67)) |
| Egyir | nr | 2013 | Ghana | Africa | 105 | 6 | 6/105 (5.7) | 6 | 6/6 (100.0) | 6/105 (5.7) | nr | Antibiotic susceptibility testing- PCR- MLST | ([69](#_ENREF_69)) |
| Champion | nr | 2011 | USA | America | 282 | 282 | 282/282 (100.0) | 14 | 14/282 (4.9) | 14/282 (4.9) | skin and soft tissues samples, umbilical cord stumps, nasal swabs, eye swabs, | Susceptibility to Antimicrobial Agents | ([70](#_ENREF_70)) |
| Brown | 1998 | 2002 | UK | Europe | 2542 | 608 | 608/2542 (23.9) | 11 | 11/608 (1.8) | 11/2542 (0.4) | lesional skin,nonlesional skin,anterior nares | Disk diffusion | ([73](#_ENREF_73)) |
| Brown | 2000 | 2002 | UK | Europe | 2893 | 1007 | 1007/2893 (34.8) | 24 | 24/1007 (2.3) | 24/2893 (0.8) | nasal | disk diffusion | ([73](#_ENREF_73)) |
| Brown | 1999 | 2002 | UK | Europe | 2883 | 889 | 889/2883 (30.8) | 8 | 8/889 (0.8) | 8/2883 (0.2) | CF patient | nr | ([73](#_ENREF_73)) |
| Brown | 2001 | 2002 | UK | Europe | 2947 | 1337 | 1337/2947 (45.3) | 27 | 27/1337 (2.0) | 27/2947 (0.9) | nr | hybridization | ([73](#_ENREF_73)) |
| Cercenado | 2004-2007 | 2008 | Spain | Europe | 13 | 13 | 13/13 (100.0) | 1 | 1/13 (7.6) | 1/13 (7.6) | skin swabs | antibiogram | ([74](#_ENREF_74)) |
| Erdenizmenli | 1999-2001 | 2004 | Turkey | Europe | 91 | 91 | 91/91 (100.0) | 17 | 17/91 (18.6) | 17/91 (18.6) | nr | nr | ([75](#_ENREF_75)) |
| McLaws | 2003-2005 | 2011 | Denmark | Europe | 1639 | 1639 | 1639/1639 (100.0) | 291 | 291/1639 (17.7) | 291/1639 (17.7) | nr | nr | ([78](#_ENREF_78)) |
| Idrees | 2005-2007 | 2009 | Pakistan | Asia | 501 | 501 | 501/501 (100.0) | 45 | 45/501 (8.9) | 45/501 (8.9) | skin and soft tissue infections, otitis , and bacteremia | E-test, PFGE, MLST | ([79](#_ENREF_79)) |
| Yu | 2012-2013 | 2015 | China | Asia | 392 | 181 | 181/392 (46.1) | 49 | 49/181 (27.0) | 49/392 (12.5) | blood samples | nr | ([80](#_ENREF_80)) |
| Rahimi | 2013 | 2016 | Iran | Asia | 491 | 491 | 491/491 (100.0) | 4 | 4/491 (0.81) | 4/491 (0.81) | urine, pus, blood, tissue, wound and ear swabs | disk diffusion | ([81](#_ENREF_81)) |
| Bari | 2013 | 2015 | Pakistan | Asia | 957 | 957 | 957/957 (100.0) | 690 | 690/957 (72.1) | 690/957 (72.1) | floor, walls, air and inanimate objects | PCR | ([82](#_ENREF_82)) |
| [Randrianirina](https://www.ncbi.nlm.nih.gov/pubmed/?term=Randrianirina%20F%5BAuthor%5D&cauthor=true&cauthor_uid=17521424) | 2001-2005 | 2007 | Madagascar | Africa | 529 | 45 | 36/529 (6.8) | 36 | 36/45(80.0) | 45/529 (8.5) | sputum, pus, catheter, blood and exudates | disk diffusion-MIC | ([85](#_ENREF_85)) |
| Cartolano | 2000 | 2004 | France | Europe | 1070 | 1070 | 1070/1070 (100.0) | 24 | 24/1070 (2.24) | 24/1070 (2.2) | nr | nr | ([87](#_ENREF_87)) |
| Coombs | 2011 | 2013 | Australia | Oceania | 2357 | 713 | 713/2357 (30.2) | 26 | 26/713 (3.6) | 26/2357 (1.1) | Pus,Blood ,Urine ,Sputum,Miscellaneous | broth microdilution/PCR | ([88](#_ENREF_88)) |
| Belabbès | 2000 | 2001 | Morocco | Africa | 345 | 88 | 88/345 (25.5) | 40 | 40/88 (45.4) | 40/345 (11.5) | nr | nr | ([93](#_ENREF_93)) |
| Huang | nr | 2013 | China | Asia | 116 | 19 | 19/116 (16.3) | 1 | 1/19 (5.2) | 1/116 (0.8) | nosocomial | disk diffusion method | ([97](#_ENREF_97)) |
| Balawi | 2014 | 2017 | Arabia | Asia | 54 | 26 | 26/54 (48.1) | 2 | 2/26 (7.6) | 2/54 (3.7) | nosocomial | disk diffusion method | ([98](#_ENREF_98)) |
| Błażewicz | 2014-2015 | 2017 | Poland | Europe | 148 | 11 | 11/148 (7.4) | 2 | 2/11 (18.1) | 2/148 (1.3) | nasal | MIC | ([99](#_ENREF_99)) |
| Jappe | 2003-2005 | 2008 | Germany | Europe | 130 | 18 | 18/130 (13.8) | 5 | 5/18 (27.7) | 5/130 (3.8) | wound sepsis infections | Bacteriology and Antibiotic Susceptibility Testing | ([102](#_ENREF_102)) |
| Jorgen | 1995-2001 | 2007 | Norway | Europe | 86 | 86 | 86/86 (100.0) | 23 | 23/86 (26.7) | 23/86 (26.7) | nr | nr | ([104](#_ENREF_104)) |
| Jorgen | 2004 | 2007 | Norway | Europe | 33 | 33 | 33/33 (100.0) | 6 | 6/33 (18.1) | 6/33 (18.1) | nr | PFGE and Southern blot | ([104](#_ENREF_104)) |
| Hwang | 2000-2002 | 2002 | Taiwan | Asia | 108 | 27 | 27/108 (25.0) | 1 | 1/27 (3.7) | 1/108 (0.9) | lesions,nares,Abscesses,Furuncles,Impetigo,Superficial skin infections, | broth microdilution | ([212](#_ENREF_212)) |
| Kareiviene | nr | 2006 | Lithuania | Europe | 294 | 17 | 17/294 (13.0) | 1 | 1/17 (5.8) | 1/294 (0.3) | method" | nr | ([213](#_ENREF_213)) |
| [Lavrentieva](https://www.researchgate.net/profile/Kateryna_Lavrentieva) | nr | 2018 | Ukraine | Europe | 335 | 169 | 169/335 (50.4) | 3 | 3/169 (1.7) | 3/335 (0.8) | nr | nr | ([214](#_ENREF_214)) |
| Kesah | 1996-1997 | 2003 | Malta | Europe | 10 | 10 | 10/10 (100.0) | 2 | 2/10 (20.0) | 2/10 (20.0) | chronic otitis media, acute otitis externa and granular myringitis | Kirby–Bauer method- | ([109](#_ENREF_109)) |
| Kesah | 1996-1997 | 2003 | Senegal | Africa | 21 | 21 | 21/21 (100.0) | 7 | 7/21 (33.3) | 7/21 (33.3) | diskharging ears | Kirby-Bauer method | ([109](#_ENREF_109)) |
| Kesah | 1996-1997 | 2003 | Morocco | Africa | 21 | 21 | 21/21 (100.0) | 11 | 11/21 (52.3) | 11/21 (52.3) | Lesional and nasal specimens | antibiotic susceptibility tests | ([109](#_ENREF_109)) |
| Kesah | 1996-1997 | 2003 | Kenya | Africa | 38 | 38 | 38/38 (100.0) | 22 | 22/38 (57.8) | 22/38 (57.8) | clinical strains | MIC | ([109](#_ENREF_109)) |
| Kesah | 1996-1997 | 2003 | Nigeria | Africa | 42 | 42 | 42/42 (100.0) | 12 | 12/42 (28.5) | 12/42 (28.5) | conjunctiva sacs, nares , groin , and anus | Antimicrobial susceptibility testing-PCR | ([109](#_ENREF_109)) |
| Kim | nr | 2009 | Korea | Asia | 20 | 20 | 20/20 (100.0) | 18 | 18/20 (90.0) | 18/20 (90.0) | lesion and no lesion agar disk-diffusion | agar disk-diffusion method. | ([111](#_ENREF_111)) |
| Lim | nr | 2013 | Malaysia | Asia | 162 | 162 | 162/162 (100.0) | 15 | 15/162 (9.2) | 15/162 (9.2) | "sputum samples | nr | ([112](#_ENREF_112)) |
| Klein | 2009–2014 | 2016 | Germany | Europe | 526 | 78 | 78/526 (14.8) | 6 | 6/78 (7.6) | 6/526 (1.1) | deep throat swab" | E. test | ([113](#_ENREF_113)) |
| Livermore | 1990 | 2002 | UK | Europe | 4800 | 82 | 82/4800 (1.7) | 1 | 1/82 (1.2) | 1/4800 (0.0) | deep throat swab" | E. test | ([117](#_ENREF_117)) |
| Livermore | 1991 | 2002 | UK | Europe | 4966 | 74 | 74/4966 (1.4) | 9 | 9/74 (12.1) | 9/4966 (0.1) | "sputum samples | nr | ([117](#_ENREF_117)) |
| Livermore | 1992 | 2002 | UK | Europe | 5049 | 131 | 131/5049 (2.5) | 10 | 10/131 (7.6) | 10/5049 (0.1) | deep throat swab | E. test | ([117](#_ENREF_117)) |
| Livermore | 1993 | 2002 | UK | Europe | 5741 | 206 | 206/5741 (3.5) | 22 | 22/206 (10.6) | 22/5741 (0.3) | sputum samples | nr | ([117](#_ENREF_117)) |
| Livermore | 1994 | 2002 | UK | Europe | 5960 | 483 | 483/5960 (8.1) | 19 | 19/483 (3.9) | 19/5960 (0.3) | deep throat swab" | E. test | ([117](#_ENREF_117)) |
| Livermore | 1995 | 2002 | UK | Europe | 6588 | 870 | 870/6588 (13.2) | 29 | 29/870 (3.3) | 29/6588 (0.4) | sputum samples | nr | ([117](#_ENREF_117)) |
| Livermore | 1996 | 2002 | UK | Europe | 7779 | 1641 | 1641/7779 (21.0) | 72 | 72/1641 (4.3) | 72/7779 (0.9) | deep throat swab | antimicrobial susceptibility tests | ([117](#_ENREF_117)) |
| Livermore | 1997 | 2002 | UK | Europe | 9328 | 2956 | 2956/9328 (31.6) | 133 | 133/2956 (4.4) | 133/9328 (1.4) | dermatology inpatients | nr | ([117](#_ENREF_117)) |
| Livermore | 1998 | 2002 | UK | Europe | 9374 | 3187 | 3187/9374 (33.9) | 118 | 118/3187 (3.7) | 118/9374 (1.2) | Eschar Muscle Blood Lung | agar dilution test and E. test | ([117](#_ENREF_117)) |
| Livermore | 1999 | 2002 | UK | Europe | 10813 | 3968 | 3968/10813 (36.6) | 159 | 159/3968 (4.0) | 159/10813 (1.4) | joint fluids /Bone and joint infections | PCR | ([117](#_ENREF_117)) |
| Livermore | 2000 | 2002 | UK | Europe | 11635 | 4886 | 4886/11635 (41.9) | 332 | 332/4886 (6.7) | 332/11635 (2.8) | skin & blood | Kirby-Bauer disk diffusion | ([117](#_ENREF_117)) |
| Livermore | 2001 | 2002 | UK | Europe | 12631 | 5431 | 5431/12631 (42.9) | 299 | 299/5431 (5.5) | 299/12631 (2.3) | Skin infection | disk diffusion | ([117](#_ENREF_117)) |
| [Razeghi](https://www.sciencedirect.com/science/article/pii/S2452014419300536#!) | nr | 2019 | Iran | Asia | 83 | 52 | 52/83 (62.6) | 3 | 3/52 (5.7) | 3/83 (3.6) | blood | nr | ([122](#_ENREF_122)) |
| Groome | 2005-2006 | 2012 | South Africa | Africa | 161 | 63 | 63/161 (39.1) | 15 | 15/63 (23.8) | 15/161 (9.3) | blood | nr | ([124](#_ENREF_124)) |
| [Sun](https://www.researchgate.net/scientific-contributions/2089395898_M_Sun) | nr | 2013 | China | Asia | 60 | 60 | 60/60 (100.0) | 2 | 2/60 (3.3) | 2/60 (3.3) | blood | nr | ([125](#_ENREF_125)) |
| Dinić | nr | 2013 | Serbia | Asia | 1381 | 123 | 123/1381 (8.9) | 4 | 4/123 (3.2) | 4/1381 (0.2) | blood | nr | ([127](#_ENREF_127)) |
| Ellington | 2002-2012 | 2015 | UK | Europe | 23 | 23 | 23/23 (100.0) | 13 | 13/23 (56.5) | 13/23 (56.5) | skin swab, and impetigo | Bacterial cultures and susceptibility testing | ([129](#_ENREF_129)) |
| Goudarzi | nr | 2020 | Iran | Asia | 120 | 120 | 120/120 (100.0) | 3 | 3/120 (2.5) | 3/120 (2.5) | skin swab | disk diffusion | ([130](#_ENREF_130)) |
| Memikoǧlu | nr | 2002 | Turkey | Europe | 225 | 225 | 225/225 (100.0) | 6 | 6/225 (2.6) | 6/225 (2.6) | nr | Kirby-Bauer disk diffusion and broth microdilution | ([131](#_ENREF_131)) |
| Khemiri | 2013 | 2017 | Libya | Africa | 32 | 32 | 32/32 (100.0) | 32 | 32/32 (100.0) | 32/32 (100.0) | skin and soft tissue | disk diffusion method (Kirby-Bauer) | ([136](#_ENREF_136)) |
| Morrissey | nr | 2004 | UK | Europe | 158 | 35 | 35/158 (22.1) | 1 | 1/35 (2.8) | 1/158 (0.6) | skin swabs | microdilution breakpoint method (VITEK 2 ) or the E. test MIC | ([137](#_ENREF_137)) |
| Nejma | 2003 to 2005 | 2008 | Tunisia | Africa | 64 | 64 | 64/64 (100.0) | 2 | 2/64 (3.1) | 2/64 (3.1) | anterior nares | disk diffusion method (Kirby-Bauer) | ([138](#_ENREF_138)) |
| Sohail | 2012-2016 | 2018 | Pakistan | Asia | 344 | 203 | 203/344 (59.0) | 43 | 43/203 (21.1) | 43/344 (12.5) | skin swabs | disk diffusion | ([139](#_ENREF_139)) |
| Ibrahim | 2012 | 2012 | Malaysia | Asia | 85 | 25 | 25/85 (29.4) | 14 | 14/25 (56.0) | 14/85 (16.4) | nr | nr | ([215](#_ENREF_215)) |
| Nergiz | 2001-2011 | 2012 | Turkey | Europe | 192 | 89 | 89/192 (46.3) | 18 | 18/89 (20.2) | 18/192 (9.3) | wound samples | Antimicrobial susceptibility of isolates-PCR | ([141](#_ENREF_141)) |
| Nickerson | nr | 2009 | Thailand | Asia | 81 | 23 | 23/81 (28.3) | 3 | 3/23 (13.0) | 3/81 (3.7) | wound,urine,tracheal aspirates,blood,cathather | disk diffusion | ([142](#_ENREF_142)) |
| Norazah | nr | 2002 | Malaysia | Asia | 640 | 640 | 640/640 (100.0) | 32 | 32/640 (5.0) | 32/640 (5.0) | dermatology patients and hospital inpatients | disk diffusion | ([145](#_ENREF_145)) |
| Norazah | 1997-1999 | 2005 | Malaysia | Asia | 685 | 685 | 685/685 (100.0) | 32 | 32/685 (4.6) | 32/685 (4.6) | clincal specimens | disk diffusion-agar dillution method | ([146](#_ENREF_146)) |
| Yıldız | nr | 2014 | Turkey | Europe | 397 | 397 | 397/397 (100.0) | 32 | 32/397 (8.0) | 32/397 (8.0) | blood, urine, wound, body fluids, and sputum | Antimicrobial Susceptibility Testing-PCR | ([147](#_ENREF_147)) |
| Pfaller | 1997-2006 | 2010 | Canada | America | 217 | 101 | 101/217 (46.5) | 7 | 7/101 (6.9) | 7/217 (3.2) | nr | nr | ([151](#_ENREF_151)) |
| [Saginur](https://www.researchgate.net/profile/Raphael_Saginur) | nr | 2006 | Canada | America | 23 | 12 | 12/23 (52.1) | 3 | 3/12 (25.0) | 3/23 (13.0) | recovered from storage | disk diffusion | ([153](#_ENREF_153)) |
| Rennie | 1995-2005 | 2006 | Canada | America | 2302 | 240 | 240/2302 (10.4) | 10 | 10/240 (4.1) | 10/2302 (0.4) | nr | nr | ([154](#_ENREF_154)) |
| Rohani | nr | 2000 | Malaysia | Asia | 390 | 155 | 155/390 (39.7) | 5 | 5/155 (3.2) | 5/390 (1.2) | nr | nr | ([158](#_ENREF_158)) |
| Lemaire | 2008 | 2011 | US | America | 94 | 94 | 94/94 (100.0) | 4 | 4/94 (4.2) | 4/94 (4.2) | nr | nr | ([161](#_ENREF_161)) |
| Saleem | nr | 2017 | Pakistan | Asia | 234 | 234 | 234/234 (100.0) | 58 | 58/234 (24.7) | nr | nr | microdilution | ([164](#_ENREF_164)) |
| Boswihi | 2016 | 2018 | Kuwait | Asia | 1327 | 1327 | 1327/1327 (100.0) | 89 | 89/1327 (6.7) | 89/1327 (6.7) | nr | agar dilution | ([166](#_ENREF_166)) |
| Perwaiz | 2004-2005 | 2007 | Pakistan | Asia | 190 | 82 | 82/190 (43.1) | 2 | 2/82 (2.4) | 2/190 (1.0) | nr | nr | ([167](#_ENREF_167)) |
| Samra | nr | 2005 | Israel | Europe | 150 | 150 | 150/150 (100.0) | 6 | 6/150 (4.0) | 6/150 (4.0) | from routine cultures | disk diffusion | ([168](#_ENREF_168)) |
| [Park](https://www.ncbi.nlm.nih.gov/pubmed/?term=Park%20SH%5BAuthor%5D&cauthor=true&cauthor_uid=26512169) | nr | 2015 | Korea | Asia | 497 | 497 | 497/497 (100.0) | 18 | 18/497 (3.6) | 18/497 (3.6) | bloodstream, respiratory tract infections, and skin | Broth microdilution | ([170](#_ENREF_170)) |
| Sasirekha | 2010-2011 | 2012 | India | Asia | 153 | 42 | 42/153 (27.4) | 28 | 28/42 (66.6) | 28/153 (18.3) | blood,urine,pus,wound,respiratory trct,cerebrospinal fluid | disk diffusion | ([171](#_ENREF_171)) |
| Scerri | nr | 2013 | Malta | Europe | 329 | 29 | 29/329 (8.81) | 23 | 23/29 (79.3) | 23/329 (6.9) | nr | nr | ([172](#_ENREF_172)) |
| Senok | nr | 2018 | Saudi Arabia | Asia | 29 | 6 | 6/29 (20.6) | 2 | 2/6 (33.3) | 2/29 (6.8) | nr | nr | ([173](#_ENREF_173)) |
| Seydi | 1996-2002 | 2004 | Senegal | Africa | 130 | 74 | 74/130 (56.9) | 3 | 3/74 (4.0) | 3/130 (2.3) | nr | disk diffusion | ([175](#_ENREF_175)) |
| Akcali | nr | 2001 | Turkey | Europe | 105 | 37 | 37/105 (35.2) | 2 | 2/37 (5.4) | 2/105 (1.9) | clinical isolates | VITEK 2 system | ([177](#_ENREF_177)) |
| Souli | 2012-2013 | 2016 | Greece | Europe | 980 | 372 | 372/980 (37.9) | 224 | 224/372 (60.2) | 224/980 (22.8) | nr | Antimicrobial susceptibility -Kirby - Bauer antibiotic testing- | ([178](#_ENREF_178)) |
| Stevens | 2005-2006 | 2006 | South Australia | Oceania | 524 | 107 | 107/524 (20.4) | 7 | 7/107 (6.5) | 7/524 (1.3) | nr | Disk diffusion | ([179](#_ENREF_179)) |
| Strandén | 2006-2010 | 2012 | Swiss | Europe | 258 | 258 | 258/258 (100.0) | 14 | 14/258 (5.4) | 14/258 (5.4) | clinical specimens | Antibiotic susceptibility testing | ([180](#_ENREF_180)) |
| Titov | 2008-2010 | 2011 | Belarus | Europe | 943 | 943 | 943/943 (100.0) | 8 | 8/943 (0.8) | 8/943 (0.8) | clinical samples | E. test and MIC | ([185](#_ENREF_185)) |
| Ertem | nr | 2013 | Turkey | Europe | 60 | 60 | 60/60 (100.0) | 5 | 5/60 (8.3) | 5/60 (8.3) | nr | nr | ([187](#_ENREF_187)) |
| Tveten | 1994-98 | 2003 | Norway | Europe | 87 | 87 | 87/87 (100.0) | 30 | 30/87 (34.4) | 30/87 (34.4) | various skin infections | agar dilution-MIC | ([188](#_ENREF_188)) |
| Udo | 2011 | 2014 | Oman | Asia | 79 | 79 | 79/79 (100.0) | 12 | 12/79 (15.1) | 12/79 (15.1) | nasal and pharyngeal | disk diffusion | ([190](#_ENREF_190)) |
| Vallières | 2000-2014 | 2015 | UK | Europe | 36 | 36 | 36/36 (100.0) | 11 | 11/36 (30.5) | 11/36 (30.5) | nr | nr | ([193](#_ENREF_193)) |
| [Gostev](https://www.researchgate.net/profile/Vladimir_Gostev) | nr | 2017 | Russia | Europe | 518 | 518 | 518/518 (100.0) | 10 | 10/518 (1.9) | 10/518 (1.9) | hospitalized patient | PCR | ([194](#_ENREF_194)) |
| Vourli | nr | 2005 | Greece | Europe | 20 | 20 | 20/20 (100.0) | 18 | 18/20 (90.0) | 18/20 (90.0) | eczema lesion | disk diffusion | ([195](#_ENREF_195)) |
| [Alfouzan](https://www.nature.com/articles/s41598-019-54794-8#auth-1) | 2016 | 2019 | Kuwait | Asia | 209 | 209 | 209/209 (100.0) | 133 | 133/209 (63.6) | 133/209 (63.6) | skin swabs | E. test | ([196](#_ENREF_196)) |
| Wang | 2006-2010 | 2015 | Taiwan | Asia | 670 | 670 | 670/670 (100.0) | 97 | 97/670 (14.4) | 97/670 (14.4) | swab of skin | KirbyBauer’s disk diffusion method | ([198](#_ENREF_198)) |
| Wiśniewska | 1990-1998 | 2000 | Poland | Europe | 225 | 225 | 225/225 (100.0) | 35 | 35/225 (15.5) | 35/225 (15.5) | clinical samples | nr | ([199](#_ENREF_199)) |
| Wisniewska | 1997-2000 | 2002 | Poland | Europe | 190 | 190 | 190/190 (100.0) | 6 | 6/190 (3.1) | 6/190 (3.1) | nasal | disk diffusion | ([200](#_ENREF_200)) |
| Liu | nr | 2017 | China | Asia | 34 | 6 | 6/34 (17.6) | 1 | 1/6 (16.6) | 1/34 (2.9) | nr | MIC | ([201](#_ENREF_201)) |
| [Chen](https://www.researchgate.net/profile/Xu_Chen151) | 2009 | 2012 | China | Asia | 85 | 47 | 47/85 (55.2) | 1 | 1/47 (2.1) | 1/85 (1.1) | nr | nr | ([202](#_ENREF_202)) |
| Huang | nr | 2013 | Taiwan | Asia | 116 | 19 | 19/116 (16.3) | 1 | 1/19 (5.2) | 1/116 (0.8) | different clinical samples | microarray | ([97](#_ENREF_97)) |
| Baek | 2010–2013 | 2016 | South Korea | Asia | 965 | 457 | 457/965 (47.3) | 223 | 223/457 (48.7) | 223/965 (23.1) | bone tissue and sinus-tract | Disk diffusion | ([205](#_ENREF_205)) |
| Huang | 2009-2011 | 2018 | Taiwan | Asia | 30 | 7 | 7/30 (23.3) | 4 | 4/7 (57.1) | 4/30 (13.3) | respiratory samples | nr | ([206](#_ENREF_206)) |
| Zinn | 1996 | 2004 | Argentina | Europe | 102 | 102 | 102/102 (100.0) | 1 | 1/102 (0.9) | 1/102 (0.9) | wound,blood,pus,sputum,respiratpry samples | disk diffusion | ([208](#_ENREF_208)) |
| Zinn | 1996 | 2004 | England | Europe | 200 | 38 | 38/200 (19.0) | 2 | 2/38 (5.2) | 2/200 (1.0) | infection sites | agar dilution method | ([208](#_ENREF_208)) |
| Zinn | 1996 | 2004 | New Zealand | Oceania | 200 | 20 | 20/200 (10.0) | 2 | 2/20 (10.0) | 2/200 (1.0) | skin infections | Antimicrobial susceptibility testing | ([208](#_ENREF_208)) |
| Zinn | 1996 | 2004 | Kuwait | Asia | 100 | 31 | 31/100 (31.0) | 2 | 2/31 (6.4) | 2/100 (2.0) | Nasal swabs | Antimicrobial Susceptibility Testing-PFGE-PCR | ([208](#_ENREF_208)) |
| Zinn | 1996 | 2004 | Greece | Europe | 100 | 63 | 63/100 (63.0) | 16 | 16/63 (25.3) | 16/100 (16.0) | Nasal | Kirby Bauer disk diffusion | ([208](#_ENREF_208)) |

MIC; Minimal Inhibitory Concentration, MBC; minimum bactericidal concentration, PCR; Polymerase Chain Reaction, PFGE; Pulsed-field gel electrophoresis, MLST; Multilocus sequence typing, nr; not report

1. Nagarajan A, Arunkumar K, Saravanan M, Sivakumar G, Krishnan P. Detection of fusidic acid resistance determinants among Staphylococcus aureus isolates causing skin and soft tissue infections from a tertiary care centre in Chennai, South India. BMC Infectious Diseases. 2012;12.

2. Akpabie A, Naga H, Giraud K, Al Rahiss R, Nadai S. [Resistance to linezolid in Staphylococcus aureus before its release]. Pathologie-biologie. 2004;52(8):493-6.

3. Osterlund A, Eden T, Olsson-Liljequist B, Haeggman S, Kahlmeter G, Swedish Study Grp Fusid A-R. Clonal spread among Swedish children of a Staphylococcus aureus strain resistant to fusidic acid. Scandinavian Journal of Infectious Diseases. 2002;34(10):729-34.

4. Larsen AR, Bocher S, Stegger A, Goering R, Pallesen LV, Skov R. Epidemiology of European community-associated methicillin-resistant Staphylococcus aureus clonal complex 80 type IV strains isolated in Denmark from 1993 to 2004. Journal of Clinical Microbiology. 2008;46(1):62-8.

5. Sarkar A, Raji A, Garaween G, Soge O, Rey-Ladino J, Al-Kattan W, et al. Antimicrobial resistance and virulence markers in methicillin sensitive Staphylococcus aureus isolates associated with nasal colonization. Microbial pathogenesis. 2016;93:8-12.

6. Senok A, Nassar R, Kaklamanos EG, Belhoul K, Abu Fanas S, Nassar M, et al. Molecular Characterization of Staphylococcus aureus Isolates Associated with Nasal Colonization and Environmental Contamination in Academic Dental Clinics. Microbial drug resistance (Larchmont, NY). 2020.

7. Senok A, Somily A, Nassar R, Garaween G, Sing GK, Müller E, et al. Emergence of novel methicillin resistant Staphylococcus aureus strains in a tertiary care facility in Riyadh, Saudi Arabia. Journal of Infection and Public Health. 2020;13(2):322.

8. Ahmadi E, Khojasteh M, Mortazavi SM, Khan-Mohammadi F, Kazemnia A, Beheshtipour J, et al. Prevalence of and risk factors for methicillin-resistant Staphylococcus aureus nasal carriage in the West of Iran: a population-based cross-sectional study. BMC Infect Dis. 2019;19(1):899.

9. Nawaz A, Razzaq A, Ijaz S, Nawaz A, Ali A, Kaleem A. Characterization of antibiotic resistant gene in Staphylococcus aureus isolated from surgical wounds. Advancements in Life Sciences. 2016;3(3):83-8.

10. Akinkunmi EO, Adesunkanmi AR, Lamikanra A. Pattern of pathogens from surgical wound infections in a Nigerian hospital and their antimicrobial susceptibility profiles. African health sciences. 2014;14(4):802-9.

11. Aktas G, Derbentli S. In vitro activity of daptomycin combinations with rifampicin, gentamicin, fosfomycin and fusidic acid against MRSA strains. Journal of Global Antimicrobial Resistance. 2017;10:223-7.

12. Al Sweih N, Mokaddas E, Jamal W, Phillips OA, Rotimi VO. In vitro activity of linezolid and other antibiotics against Gram-positive bacteria from the major teaching hospitals in Kuwait. Journal of chemotherapy (Florence, Italy). 2005;17(6):607-13.

13. Abouelfetouh A, Kassem M, Naguib M, El-Nakeeb M. Investigation and Treatment of Fusidic Acid Resistance Among Methicillin-Resistant Staphylococcal Isolates from Egypt. Microb Drug Resist. 2017;23(1):8-17.

14. Aldasouqi R, Abu-Qatouseh L, Badran E, Alhaj Mahmoud S, Darwish M. Genetic determinants of resistance to fusidic acid among staphylococcus aureus isolates in Jordan. Jundishapur Journal of Microbiology. 2019;12(3).

15. Somily AM, Peaper DR, Paintsil E, Murray TS. Comparison of Disk Diffusion and Etest Methods to Determine the Susceptibility of Staphylococcus aureus Circulating in Riyadh, Saudi Arabia to Fusidic Acid. International journal of microbiology. 2012;2012:391251.

16. Hasani A, Sheikhalizadeh V, Hasani A, Naghili B, Valizadeh V, Nikoonijad AR. Methicillin resistant and susceptible Staphylococcus aureus: Appraising therapeutic approaches in the Northwest of Iran. Iranian Journal of Microbiology. 2013;5(1):56-62.

17. Al-Talib H, Al-Khateeb A, Hassan H. Antimicrobial resistance of Staphylococcus aureus isolates in Malaysian tertiary hospital. International Medical Journal. 2015;22(2):73-5.

18. Aqel AA, Alzoubi HM, Vickers A, Pichon B, Kearns AM. Molecular epidemiology of nasal isolates of methicillin-resistant Staphylococcus aureus from Jordan. Journal of Infection and Public Health. 2015;8(1):90-7.

19. Cabrera A, Golding G, Campbell J, Pelude L, Bryce E, Frenette C, et al. Characterization of clinical methicillin-resistant staphylococcus aureus (MRSA) isolates from Canadian hospitals, 2010-2015. Open Forum Infectious Diseases. 2016;3.

20. Vindel A, Trincado P, Cuevas O, Ballesteros C, Bouza E, Cercenado E. Molecular epidemiology of community-associated methicillin-resistant Staphylococcus aureus in Spain: 2004-12. Journal of Antimicrobial Chemotherapy. 2014;69(11):2913-9.

21. Doudoulakakis A, Spiliopoulou I, Spyridis N, Giormezis N, Kopsidas J, Militsopoulou M, et al. Emergence of a Staphylococcus aureus Clone Resistant to Mupirocin and Fusidic Acid Carrying Exotoxin Genes and Causing Mainly Skin Infections. J Clin Microbiol. 2017;55(8):2529-37.

22. Shore AC, Brennan OM, Deasy EC, Rossney AS, Kinnevey PM, Ehricht R, et al. DNA microarray profiling of a diverse collection of nosocomial methicillin-resistant Staphylococcus aureus isolates assigns the majority to the correct sequence type and staphylococcal cassette chromosome mec (SCCmec) type and results in the subsequent identification and characterization of novel SCCmec-SCCM1 composite islands. Antimicrobial Agents and Chemotherapy. 2012;56(10):5340-55.

23. Atmaca S, Özekinci T, Özerdem N. Fusidic acid susceptibilities of methicillin-sensitive and methicillin-resistant staphylococcus aureus strains. Mikrobiyoloji Bulteni. 2001;35(1):25-8.

24. Baek YS, Song HJ. Fusidic acid and mupirocin resistance of Staphylococcus aureus isolated from infected skin wounds of Korean patients. Journal of Dermatology. 2012;39:21-2.

25. Ahmad B, Khan F, Ahmed J, Cha SB, Shin MK, Bashir S, et al. Antibiotic resistance pattern and molecular epidemiology of methicillin-resistant Staphylococcus aureus colonization in burns unit of a tertiary care hospital in Peshawar, Pakistan. Tropical Journal of Pharmaceutical Research. 2014;13(12):2091-9.

26. Bauer CC, Apfalter P, Daxboeck F, Bachhofner N, Stadler M, Blacky A, et al. Prevalence of panton-valentine leukocidin genes in methicillin-resistant staphylococcus aureus isolates phenotypically consistent with community-acquired MRSA, 1999-2007, vienna general hospital. European Journal of Clinical Microbiology and Infectious Diseases. 2009;28(8):909-12.

27. Berktold M, Grif K, Maser M, Witte W, Wurzner R, Orth-Holler D. Genetic characterization of Panton-Valentine leukocidin-producing methicillin-resistant Staphylococcus aureus in Western Austria. Wiener klinische Wochenschrift. 2012;124(19-20):709-15.

28. Bernard P, Jarlier V, Santerre-Henriksen A. Antibiotic susceptibility of Staphylococcus aureus strains responsible for cutaneous infections in the community. Annales de Dermatologie et de Venereologie. 2008;135(1):13-9.

29. Bessa GR, Machado DC, Weber MB, D’Azevedo PA, Quinto VP, Lipnharski C, et al. Staphylococcus aureus resistance to topical antimicrobials in atopic dermatitis. Anais Brasileiros de Dermatologia. 2016;91(5):604-10.

30. Boswihi SS, Udo EE, Al-Sweih N. Shifts in the clonal distribution of methicillin-resistant staphylococcus aureus in Kuwait hospitals: 1992-2010. PLoS ONE. 2016;11(9).

31. Brosnikoff C, Rennie R, Kidson P, Yamamura D, Bechard C, Kelly M, et al. Surveillance of staphylococcus aureus susceptibility to fusidic acid in five Canadian laboratories. International Journal of Antimicrobial Agents. 2009;34:S43.

32. Budimir A, Tićac B, Rukavina T, Farkaš M, Kalenić S. First report on PVL-positive methicillin-resistant Staphylococcus aureus of SCCmec type V, spa type T441 in Croatia. Collegium antropologicum. 2016;40(2):133-7.

33. Budri PE, Shore AC, Coleman DC, Kinnevey PM, Humpreys H, Fitzgerald-Hughes D. Observational cross-sectional study of nasal staphylococcal species of medical students of diverse geographical origin, prior to healthcare exposure: prevalence of SCCmec, fusC, fusB and the arginine catabolite mobile element (ACME) in the absence of selective antibiotic pressure. Bmj Open. 2018;8(4).

34. Horner C, Kearns A, Heritage J, Wilcox M. The epidemiology of methicillin-resistant Staphylococcus aureus in elderly residents of 65 care homes in a single primary care trust of northern England. Clinical Microbiology and Infection. 2012;18:341.

35. Quentin C, Grobost F, Fischer I, Dutilh B, Brochet JP, Jullin J, et al. Antibiotic resistance of Staphylococcus aureus in extra-hospital practice: A six-month period study in aquitaine. Pathologie Biologie. 2001;49(1):33-40.

36. Claesson C, Nilsson LE, Kronvall G, Walder M, Sorberg M. Antimicrobial activity of tigecycline and comparative agents against clinical isolates of staphylococci and enterococci from ICUs and general hospital wards at three Swedish university hospitals. Scand J Infect Dis. 2009;41(3):171-81.

37. Castanheira M, Farrell D, Janechek M, Jones R. CEM-102 (fusidic acid) in vitro activity and evaluation of molecular resistance mechanisms among European Gram-positive isolates, 2008-2009. Clinical Microbiology and Infection. 2010;16:S247-S8.

38. Castanheira M, Watters AA, Bell JM, Turnidge JD, Jones RN. Fusidic acid resistance rates and prevalence of resistance mechanisms among Staphylococcus spp. isolated in North America and Australia, 2007-2008. Antimicrob Agents Chemother. 2010;54(9):3614-7.

39. Çetinkol Y, Aladal M, Ünal N, Bayramoglu G, Ahmetbayik S, Dogan M, et al. Comparison of fusidic acid susceptibility of staphylococci: A multicenter study. Acta Medica Mediterranea. 2018;34(2):397-402.

40. Chen HJ, Hung WC, Tseng SP, Tsai JC, Hsueh PR, Teng LJ. Fusidic acid resistance determinants in Staphylococcus aureus clinical isolates. Antimicrob Agents Chemother. 2010;54(12):4985-91.

41. Chen CM, Huang M, Chen HF, Ke SC, Li CR, Wang JH, et al. Fusidic acid resistance among clinical isolates of methicillin-resistant Staphylococcus aureus in a Taiwanese hospital. BMC microbiology. 2011;11:98.

42. Chuamuangphan T, Chongtrakool P, Sungkanuparph S. Predicting factors of methicillin-resistant Staphylococcus aureus bacteremia among hospitalized patients in a tertiary-care hospital. International Journal of Antimicrobial Agents. 2013;42:S147.

43. Cirkovic I, Svabic Vlahovic M, Stepanovic S. Molecular characterization of Panton-Valentine leukocidin positive methicillin-resistant Staphylococcus aureus isolates in Serbia. Clinical Microbiology and Infection. 2010;16:S278.

44. Yeung CK, Chow WC, Chan HHL, Ho PL. Carriage of antibiotic-resistant Staphylococcus aureus in atopic dermatitis children attending paediatric outpatient clinics. Hong Kong Journal of Dermatology and Venereology. 2010;18(3):125-31.

45. Conceicao T, Martins H, Rodrigues S, de Lencastre H, Aires-de-Sousa M. Staphylococcus aureus nasal carriage among homeless population in Lisbon, Portugal. European journal of clinical microbiology & infectious diseases : official publication of the European Society of Clinical Microbiology. 2019;38(11):2037-44.

46. Coşkun MV, Alper Y, Uyanık MH, Yazgı H. Sensitivity of methicillin-resistant staphylococcus aureus strains to fusidic acid and other non-β-lactam antibiotics. Klimik Dergisi. 2019;32(1):52-6.

47. Sahm DF, Deane J, Pillar CM, Fernandes P. In vitro activity of CEM-102 (fusidic acid) against prevalent clones and resistant phenotypes of Staphylococcus aureus. Antimicrob Agents Chemother. 2013;57(9):4535-6.

48. Davey RX, Tong SYC. The epidemiology of Staphylococcus aureus skin and soft tissue infection in the southern Barkly region of Australia's Northern Territory in 2017. Pathology. 2019;51(3):308-12.

49. Farrell DJ, Mendes RE, Castanheira M, Jones RN. Activity of Fusidic Acid Tested against Staphylococci Isolated from Patients in U.S. Medical Centers in 2014. Antimicrob Agents Chemother. 2016;60(6):3827-31.

50. Decousser JW, Desroches M, Bourgeois-Nicolaos N, Potier J, Jehl F, Lina G, et al. Susceptibility trends including emergence of linezolid resistance among coagulase-negative staphylococci and meticillin-resistant Staphylococcus aureus from invasive infections. International Journal of Antimicrobial Agents. 2015;46(6):622-30.

51. Demir T, Coplu N, Bayrak H, Turan M, Buyukguclu T, Aksu N, et al. Panton-Valentine leucocidin gene carriage among Staphylococcus aureus strains recovered from skin and soft tissue infections in Turkey. The Journal of antimicrobial chemotherapy. 2012;67(4):837-40.

52. den Heijer CD, van Bijnen EM, Paget WJ, Stobberingh EE. Fusidic acid resistance in Staphylococcus aureus nasal carriage strains in nine European countries. Future microbiology. 2014;9(6):737-45.

53. Denis O, Deplano A, De Beenhouwer H, Hallin M, Huysmans G, Garrino MG, et al. Polyclonal emergence and importation of community-acquired methicillin-resistant Staphylococcus aureus strains harbouring Panton-Valentine leucocidin genes in Belgium. The Journal of antimicrobial chemotherapy. 2005;56(6):1103-6.

54. Ghaith DM, Elnagdy SM, Tharwat NA, Said M. Expression of FusC gene Among methicillin resistant Staph aureus clinical isolates resistant to fusidic acid, Cairo, Egypt. Infectious Diseases. 2019;51(11-12):878-9.

55. Anastasiou E, Farmaki EE, Pertsas E, Kakasi E, Koteli A. In vitro activity of fusidic acid against Staphylococcus aureus strains. International Journal of Infectious Diseases. 2011;15:S18.

56. Lindberg E, Adlerberth I, Wold AE. Antibiotic resistance in Staphylococcus aureus colonising the intestines of Swedish infants. Clinical microbiology and infection : the official publication of the European Society of Clinical Microbiology and Infectious Diseases. 2004;10(10):890-4.

57. Udo E, Al-sweih N. Emergence and characterisation of community-associated Methicillin-resistant Staphylococcus aureus at a neonatal special care unit. International Journal of Infectious Diseases. 2012;16:e387.

58. Udo E, AlFouzan W, Boloki H, Musaileem WA, Verghese T. Investigation of Fusidic Acid Resistance Determinants in Methicillin-Resistant Staphylococcus aureus obtained in Kuwait Hospitals. Journal of Infection and Public Health. 2020;13(2):324.

59. Champion MD, Gray V, Eberhard C, Kumar S. The Evolutionary History of Amino Acid Variations Mediating Increased Resistance of S-aureus Identifies Reversion Mutations in Metabolic Regulators. Plos One. 2013;8(2).

60. Udo EE, Al-Sweih N, Mohanakrishnan S, West PW. Antibacterial resistance and molecular typing of methicillin-resistant Staphylococcus aureus in a Kuwaiti general hospital. Medical principles and practice : international journal of the Kuwait University, Health Science Centre. 2006;15(1):39-45.

61. Udo EE, Sarkhoo E. The dissemination of ST80-SCCmec-IV community-associated methicillin resistant Staphylococcus aureus clone in Kuwait hospitals. Annals of Clinical Microbiology and Antimicrobials. 2010;9.

62. Udo EE, Al-Sweih N, Mokaddas E, Johny M, Dhar R, Gomaa HH, et al. Antibacterial resistance and their genetic location in MRSA isolated in Kuwait hospitals, 1994-2004. BMC Infect Dis. 2006;6:168.

63. Udo EE, Al-Sweih N. Emergence of methicillin-resistant Staphylococcus aureus in the Maternity Hospital, Kuwait. Medical principles and practice : international journal of the Kuwait University, Health Science Centre. 2013;22:535-9.

64. Udo EE, Al-Sweih N. Dominance of community-associated methicillin-resistant Staphylococcus aureus clones in a maternity hospital. PLoS ONE. 2017;12(6).

65. Udo EE, Boswihi SS. Antibiotic Resistance Trends in Methicillin-Resistant Staphylococcus aureus Isolated in Kuwait Hospitals: 2011-2015. Medical principles and practice : international journal of the Kuwait University, Health Science Centre. 2017;26(5):485-90.

66. Udo E, Sarkhoo E. The expansion of ST80-SCCmec-IV clone of communityacquired methicillin resistant Staphylococcus aureus in Kuwait hospitals. International Journal of Infectious Diseases. 2010;14:e345-e6.

67. Udo EE, Panigrahi D, Jamsheer AE. Molecular typing of methicillin-resistant Staphylococcus aureus isolated in a Bahrain hospital. Medical principles and practice : international journal of the Kuwait University, Health Science Centre. 2008;17(4):308-14.

68. Edslev SM, Clausen ML, Agner T, Stegger M, Andersen PS. Genomic analysis reveals different mechanisms of fusidic acid resistance in Staphylococcus aureus from Danish atopic dermatitis patients. The Journal of antimicrobial chemotherapy. 2018;73(4):856-61.

69. Egyir B, Guardabassi L, Nielsen SS, Larsen J, Addo KK, Newman MJ, et al. Prevalence of nasal carriage and diversity of Staphylococcus aureus among inpatients and hospital staff at Korle Bu Teaching Hospital, Ghana. Journal of Global Antimicrobial Resistance. 2013;1(4):189-93.

70. Champion EA, Popowitch E, Miller M, Saiman L, Muhlebach M. MRSA: Epidemiology, molecular typing and antimicrobial susceptibilities: Multicenter STAR-CF study. American Journal of Respiratory and Critical Care Medicine. 2011;183(1).

71. Scicluna EA, Shore A, Thürmer A, Slickers P, Ehricht R, Borg MA, et al. Molecular epidemiology of MRSA in Malta and the description of a Maltese epidemic MRSA strain. International Journal of Medical Microbiology. 2009;299:40-1.

72. El-Zimaity D, Kearns AM, Dawson SJ, Price S, Harrison GAJ. Survey, characterization and susceptibility to fusidic acid of Staphylococcus aureus in the Carmarthen area. Journal of Antimicrobial Chemotherapy. 2004;54(2):441-6.

73. Brown EM, Thomas P. Fusidic acid resistance in Staphylococcus aureus isolates. Lancet (London, England). 2002;359(9308):803.

74. Cercenado E, Cuevas O, Marin M, Bouza E, Trincado P, Boquete T, et al. Community-acquired methicillin-resistant Staphylococcus aureus in Madrid, Spain: transcontinental importation and polyclonal emergence of Panton-Valentine leukocidin-positive isolates. Diagnostic microbiology and infectious disease. 2008;61(2):143-9.

75. Erdenizmenli M, Yapar N, Sengonul A, Yuce A, Cakir N, Yulug N. In-vitro activity of fusidic acid against methicillin-resistant Staphylococcus aureus. Journal of chemotherapy (Florence, Italy). 2004;16(3):310-1.

76. Hanif E, Hassan SA. Evaluation of antibiotic resistance pattern in clinical isolates of Staphylococcus aureus. Pakistan journal of pharmaceutical sciences. 2019;32(3 (Supplementary)):1219-23.

77. Khan F, Ahmad B, Ahmed J, Yoo HS, Bashir S. Investigation of Staphylococcus aureus, prevailing in the environment of Khyber Teaching Hospital, Peshawar, Pakistan. Pakistan journal of pharmaceutical sciences. 2018;31(2):429-37.

78. McLaws FB, Larsen AR, Skov RL, Chopra I, O'Neill AJ. Distribution of fusidic acid resistance determinants in methicillin-resistant Staphylococcus aureus. Antimicrob Agents Chemother. 2011;55(3):1173-6.

79. Idrees F, Jabeen K, Khan MS, Zafar A. Antimicrobial resistance profile of methicillin resistant staphylococcal aureus from skin and soft tissue isolates. JPMA The Journal of the Pakistan Medical Association. 2009;59(5):266-9.

80. Yu FY, Liu YL, Lu CH, Lv JN, Qi XQ, Ding Y, et al. Dissemination of fusidic acid resistance among Staphylococcus aureus clinical isolates. BMC microbiology. 2015;15.

81. Rahimi F, Shokoohizadeh L. Characterization of methicillin resistant Staphylococcus aureus strains among inpatients and outpatients in a referral hospital in Tehran, Iran. Microbial pathogenesis. 2016;97:89-93.

82. Bari F, Wazir R, Haroon M, Ali S, Imtiaz, Rahman H, et al. FREQUENCY AND ANTIBIOTIC SUSCEPTIBILITY PROFILE OF MRSA AT LADY READING HOSPITAL, PESHAWAR. Gomal Journal of Medical Sciences. 2015;13(1):62-5.

83. Fong IL, Razak E, Mei JTJ, Safian NAB, Tian OS, Peng NP, et al. Prevalence and antibiotic sensitivity profiles of Staphylococcus aureus nasal carriage among preclinical and clinical medical students in a Malaysian university. Malaysian Journal of Microbiology. 2018;14(4):351-5.

84. Schaefer F, Bruttin O, Zografos L, Guex-Crosier Y. Bacterial keratitis: a prospective clinical and microbiological study. The British journal of ophthalmology. 2001;85(7):842-7.

85. Randrianirina F, Soares JL, Ratsima E, Carod JF, Combe P, Grosjean P, et al. In vitro activities of 18 antimicrobial agents against Staphylococcus aureus isolates from the Institut Pasteur of Madagascar. Ann Clin Microbiol Antimicrob. 2007;6:5.

86. Frickmann H, Hahn A, Berlec S, Ulrich J, Jansson M, Schwarz NG, et al. On the Etiological Relevance of Escherichia coli and Staphylococcus aureus in Superficial and Deep Infections-A Hypothesis-Forming, Retrospective Assessment. European Journal of Microbiology and Immunology. 2019;9(4):124-30.

87. Cartolano GL, Cheron M, Benabid D, Leneveu M, Boisivon A, Marchal MF, et al. Methicillin-resistant Staphylococcus aureus (MRSA) with reduced susceptibility to glycopeptides (GISA) in 63 French general hospitals. Clinical Microbiology and Infection. 2004;10(5):448-51.

88. Coombs GW, Nimmo GR, Pearson JC, Collignon PJ, Bell JM, McLaws ML, et al. Australian Group on Antimicrobial Resistance Hospital-onset Staphylococcus aureus Surveillance Programme annual report, 2011. Communicable diseases intelligence quarterly report. 2013;37(3):E210-8.

89. Gilani SJK, Gonzalez M, Hussain I, Finlay AY, Patel GK. Staphylococcus aureus re-colonization in atopic dermatitis: beyond the skin. Clinical and Experimental Dermatology. 2005;30(1):10-3.

90. Gordon NC, Price JR, Cole K, Everitt R, Morgan M, Finney J, et al. Prediction of Staphylococcus aureus antimicrobial resistance by whole-genome sequencing. J Clin Microbiol. 2014;52(4):1182-91.

91. Gosbell IB, Mercer JL, Neville SA, Chant KG, Munro R. Community-acquired, non-multiresistant oxacillin-resistant Staphylococcus aureus (NORSA) in South Western Sydney. Pathology. 2001;33(2):206-10.

92. Gubbay JB, Gosbell IB, Barbagiannakos T, Vickery AM, Mercer JL, Watson M. Clinical features, epidemiology, antimicrobial resistance, and exotoxin genes (including that of Panton-Valentine leukocidin) of gentamicin-susceptible methicillin-resistant Staphylococcus aureus (GS-MRSA) isolated at a paediatric teaching hospital in New South Wales, Australia. Pathology. 2008;40(1):64-71.

93. Belabbes H, Elmdaghri N, Hachimi K, Marih L, Zerouali K, Benbachir M. Antibiotic resistance of Staphylococcus aureus isolated from community and nosocomial infections in Casablanca. Medecine Et Maladies Infectieuses. 2001;31(1):25-8.

94. Harkins CP, McAleer MA, Fleury OM, Bennett D, Foster TJ, McLean WHI, et al. Staphylococcus aureus associated with atopic eczema flares: Case-control study. British Journal of Dermatology. 2016;174(5):e43.

95. Heng YK, Tan KT, Sen P, Chow A, Leo YS, Lye DC, et al. Staphylococcus aureus and topical fusidic acid use: Results of a clinical audit on antimicrobial resistance. International Journal of Dermatology. 2013;52(7):876-81.

96. Harastani HH, Tokajian ST. Community-associated methicillin-resistant Staphylococcus aureus clonal complex 80 type IV (CC80-MRSA-IV) isolated from the Middle East: a heterogeneous expanding clonal lineage. PLoS One. 2014;9(7):e103715.

97. Huang YC, Su LH, Lin TY. Nasal carriage of methicillin-resistant Staphylococcus aureus among pediatricians in Taiwan. PLoS ONE. 2013;8(11).

98. Al Balawi I, Amirthalingam P, Alyoussef AAK, Mohammed OS, Mirghani HO, Ezzat AA. Antibiotic susceptibility pattern of methicillin-resistant Staphylococcus aureus from the isolated wound culture in the northwest region, Kingdom of Saudi Arabia. Asian Journal of Pharmaceutical Research and Health Care. 2017;9(1):1-6.

99. Błazewicz I, Jaśkiewicz M, Bauer M, Piechowicz L, Nowicki RJ, Kamysz W, et al. Decolonization of Staphylococcus aureus in patients with atopic dermatitis: A reason for increasing resistance to antibiotics? Postepy Dermatologii i Alergologii. 2017;34(6):553-60.

100. Wang JT, Huang IW, Chang SC, Tan MC, Lai JF, Chen PY, et al. Increasing resistance to fusidic acid among clinical isolates of MRSA. The Journal of antimicrobial chemotherapy. 2017;72(2):616-8.

101. Li J, Wang Q, Wang L, Sun M, Li S, Sun J, et al. Multidrug-resistant clones of methicillin-resistant Staphylococcus aureus isolated from Chinese children with community-associated pneumonia. Chinese Journal of Infection and Chemotherapy. 2014;14(1):32-7.

102. Jappe U, Heuck D, Strommenger B, Wendt C, Werner G, Altmann D, et al. Staphylococcus aureus in dermatology outpatients with special emphasis on community-associated methicillin-resistant strains. The Journal of investigative dermatology. 2008;128(11):2655-64.

103. Jones RN, Castanheira M, Rhomberg PR, Woosley LN, Pfaller MA. Performance of fusidic acid (CEM-102) susceptibility testing reagents: Broth microdilution, disk diffusion, and Etest methods as applied to Staphylococcus aureus. Journal of Clinical Microbiology. 2010;48(3):972-6.

104. Jorgen B, Merckoll P, Melby KK. Susceptibility to daptomycin, quinupristin-dalfopristin and linezolid and some other antibiotics in clinical isolates of methicillin resistant and methicillin sensitive S.aureus from the Oslo area. Scand J Infect Dis. 2007;39(11-12):1059-62.

105. Hwang JH, Chu CK, Liu TC. Changes in bacteriology of diskharging ears. The Journal of laryngology and otology. 2002;116(9):686-9.

106. Park JM, Jo JH, Jin H, Ko HC, Kim MB, Kim JM, et al. Change in Antimicrobial Susceptibility of Skin-Colonizing Staphylococcus aureus in Korean Patients with Atopic Dermatitis during Ten-Year Period. Annals of Dermatology. 2016;28(4):470-8.

107. Bierowiec K, Ploneczka-Janeczko K, Rypula K. Is the Colonisation of Staphylococcus aureus in Pets Associated with Their Close Contact with Owners? Plos One. 2016;11(5).

108. Kedzierska A, Kapinska-Mrowiecka M, Czubak-Macugowska M, Wojcik K, Kedzierska J. Susceptibility testing and resistance phenotype detection in Staphylococcus aureus strains isolated from patients with atopic dermatitis, with apparent and recurrent skin colonization. The British journal of dermatology. 2008;159(6):1290-9.

109. Kesah C, Ben Redjeb S, Odugbemi TO, Boye CS, Dosso M, Ndinya Achola JO, et al. Prevalence of methicillin-resistant Staphylococcus aureus in eight African hospitals and Malta. Clinical microbiology and infection : the official publication of the European Society of Clinical Microbiology and Infectious Diseases. 2003;9(2):153-6.

110. Kim MS, Chung BS, Choi KC. A study of antibiotic susceptibility of Staphylococcus aureus in bacterial skin infections. Korean Journal of Dermatology. 2006;44(7):805-10.

111. Kim SM, Lee DC, Park SD, Kim BS, Kim JK, Choi MR, et al. Genotype, coagulase type and antimicrobial susceptibility of methicillin-resistant Staphylococcus aureus isolated from dermatology patients and healthy individuals in Korea. Journal of Bacteriology and Virology. 2009;39(4):307-16.

112. Lim KT, Abu Hanifah Y, Yusof MYM, Ito T, Thong KL. Comparison of methicillin-resistant Staphylococcus aureus strains isolated in 2003 and 2008 with an emergence of multidrug resistant ST22: SCCmec IV clone in a tertiary hospital, Malaysia. Journal of Microbiology Immunology and Infection. 2013;46(3):224-33.

113. Klein S, Nurjadi D, Eigenbrod T, Bode KA. Evaluation of antibiotic resistance to orally administrable antibiotics in staphylococcal bone and joint infections in one of the largest university hospitals in Germany: is there a role for fusidic acid? Int J Antimicrob Agents. 2016;47(2):155-7.

114. Kunsang Bhutia O, Singh TSK. Occurrence and antimicrobial susceptibility pattern of community and hospital associated methicillin resistant staphylococcus aureus strains in Sikkim. Journal International Medical Sciences Academy. 2012;25(4):235-7.

115. Laurent F, Tristan A, Croze M, Bes M, Meugnier H, Lina G, et al. Presence of the epidemic European fusidic acid-resistant impetigo clone (EEFIC) of Staphylococcus aureus in France. The Journal of antimicrobial chemotherapy. 2009;63(2):420-1; author reply 1.

116. Lescat M, Dupeyron C, Faubert E, Mangeney N. Pulse-field gel electrophoresis typing of methicillin-resistant Staphylococcus aureus strains susceptible to aminoglycosides isolated from 1993 to 2002. The Journal of hospital infection. 2004;57(3):253-7.

117. Livermore D, James D, Duckworth G, Stephens P. Fusidic-acid use and resistance. Lancet (London, England). 2002;360(9335):806.

118. Lorette G, Beaulieu P, Bismuth R, Duru G, Guihard W, Lemaitre M, et al. Community-acquired cutaneous infections: Causal role of some bacteria and sensitivity to antibiotics. Annales de Dermatologie et de Venereologie. 2003;130(8-9 I):723-8.

119. Salah LA, Faergemann J. A retrospective analysis of skin bacterial colonisation, susceptibility and resistance in atopic dermatitis and impetigo patients. Acta dermato-venereologica. 2015;95(5):532-5.

120. Shah M, Mohanraj M. High levels of fusidic acid-resistant Staphylococcus aureus in dermatology patients. The British journal of dermatology. 2003;148(5):1018-20.

121. Alreshidi MA, Mariana NS. Increasing rate of detection of fusidic acid resistance in methicillin-resistant Staphylococcus aureus isolated from clinical samples in Malaysia. The Medical journal of Malaysia. 2011;66(3):276.

122. Razeghi M, Saffarian P, Goudarzi M. Incidence of inducible clindamycin resistance and antibacterial resistance genes variability in clinical Staphylococcus aureus strains: A two-year multicenter study in Tehran, Iran. Gene Reports. 2019;16.

123. Denton M, O'Connell B, Bernard P, Jarlier V, Williams Z, Henriksen AS. The EPISA study: antimicrobial susceptibility of Staphylococcus aureus causing primary or secondary skin and soft tissue infections in the community in France, the UK and Ireland. The Journal of antimicrobial chemotherapy. 2008;61(3):586-8.

124. Groome MJ, Albrich WC, Wadula J, Khoosal M, Madhi SA. Community-onset Staphylococcus aureus bacteraemia in hospitalised African children: high incidence in HIV-infected children and high prevalence of multidrug resistance. Paediatrics and international child health. 2012;32(3):140-6.

125. Sun M, Wang L, Liu Y, Li X, Sun J, Wang C, et al. Molecular characterization and antimicrobial resistance of community-associated methicillin-resistant Staphylococcus aureus strains isolated from children with skin and soft tissue infections. Chinese Journal of Infection and Chemotherapy. 2013;13(1):19-24.

126. Niebuhr M, Mai U, Kapp A, Werfel T. Antibiotic treatment of cutaneous infections with Staphylococcus aureus in patients with atopic dermatitis: current antimicrobial resistances and susceptibilities. Experimental dermatology. 2008;17(11):953-7.

127. Dinić M, Vuković S, Kocić B, Dordević DS, Bogdanović M. Nasal carriage of Staphylococcus aureus in healthy adults and in school children. Acta Facultatis Medicae Naissensis. 2013;30(1):31-6.

128. Abdallah M, Zaki SM, El-Sayed A, Erfan D. Evaluation of secondary bacterial infection of skin diseases in Egyptian in-and out-patients and their sensitivity to antimicrobials. Egyptian Dermatol Online J. 2007;3:1-15.

129. Ellington MJ, Reuter S, Harris SR, Holden MTG, Cartwright EJ, Greaves D, et al. Emergent and evolving antimicrobial resistance cassettes in community-associated fusidic acid and meticillin-resistant Staphylococcus aureus. International Journal of Antimicrobial Agents. 2015;45(5):477-84.

130. Goudarzi M, Tayebi Z, Dadashi M, Miri M, Amirpour A, Fazeli M. Characteristics of community-acquired methicillin-resistant Staphylococcus aureus associated with wound infections in Tehran, Iran: High prevalence of PVL+ t008 and the emergence of new spa types t657, t5348, and t437 in Iran. Gene Reports. 2020;19.

131. Memikoǧlu KO, Bayar B, Kurt Ö, Çokça F. In-vitro susceptibility of methicillin resistant Staphylococcus aureus to fusidic acid and trimethoprim - Sulfamethoxazole. Mikrobiyoloji Bulteni. 2002;36(2):141-5.

132. Alsterholm M, Flytstrom I, Bergbrant IM, Faergemann J. Fusidic acid-resistant Staphylococcus aureus in impetigo contagiosa and secondarily infected atopic dermatitis. Acta dermato-venereologica. 2010;90(1):52-7.

133. Mitra A, Mohanraj M, Shah M. High levels of fusidic acid-resistant Staphylococcus aureus despite restrictions on antibiotic use. Clin Exp Dermatol. 2009;34(2):136-9.

134. Elazhari M, Zerouali K, Dersi N, Saile R, Timinouni M, Hassar M. Variability of fusidic acid-resistant methicillin-sensitive Staphylococcus aureus isolates in Casablanca, Morocco. Clinical Microbiology and Infection. 2010;16:S281.

135. Shahmohammadi MR, Nahaei MR, Akbarzadeh A, Milani M. Clinical test to detect mecA and antibiotic resistance in Staphylococcus aureus, based on novel biotechnological methods. Artificial cells, nanomedicine, and biotechnology. 2016;44(6):1464-8.

136. Khemiri M, Akrout Alhusain A, Abbassi MS, El Ghaieb H, Santos Costa S, Belas A, et al. Clonal spread of methicillin-resistant Staphylococcus aureus-t6065-CC5-SCCmecV-agrII in a Libyan hospital. Journal of Global Antimicrobial Resistance. 2017;10:101-5.

137. Morrissey I, Burnett R, Viljoen L, Robbins M. Surveillance of the susceptibility of ocular bacterial pathogens to the fluoroquinolone gatifloxacin and other antimicrobials in Europe during 2001/2002. The Journal of infection. 2004;49(2):109-14.

138. Ben Nejma M, Mastouri M, Bel Hadj Jrad B, Nour M. Characterization of ST80 Panton-Valentine leukocidin-positive community-acquired methicillin-resistant Staphylococcus aureus clone in Tunisia. Diagnostic microbiology and infectious disease. 2013;77(1):20-4.

139. Sohail M, Latif Z. Molecular analysis, biofilm formation, and susceptibility of methicillin-resistant Staphylococcus aureus strains causing community-and health care-associated infections in central venous catheters. Revista da Sociedade Brasileira de Medicina Tropical. 2018;51(5):603-9.

140. Woodford N, Afzal-Shah M, Warner M, Livermore DM. In vitro activity of retapamulin against Staphylococcus aureus isolates resistant to fusidic acid and mupirocin. The Journal of antimicrobial chemotherapy. 2008;62(4):766-8.

141. Nergiz Ş, Atmaca S, Özekinci T, Tekin A. Fusidic acid resistance in staphylococcus aureus strains in an interval of ten years (2001-2011). Turkiye Klinikleri Journal of Medical Sciences. 2012;32(6):1668-72.

142. Nickerson EK, Hongsuwan M, Limmathurotsakul D, Wuthiekanun V, Shah KR, Srisomang P, et al. Staphylococcus aureus bacteraemia in a tropical setting: patient outcome and impact of antibiotic resistance. PLoS One. 2009;4(1):e4308.

143. Scangarella-Oman NE, Shawar RM, Bouchillon S, Hoban D. Microbiological profile of a new topical antibacterial: retapamulin ointment 1%. Expert review of anti-infective therapy. 2009;7(3):269-79.

144. Norazah A, Lim VK, Munirah SN, Kamel AG. Staphylococcus aureus carriage in selected communities and their antibiotic susceptibility patterns. The Medical journal of Malaysia. 2003;58(2):255-61.

145. Norazah A, Lim VKE, Koh YT, Rohani MY, Zuridah H, Spencer K, et al. Molecular fingerprinting of fusidic acid- and rifampicin-resistant strains of methicillin-resistant Staphylococcus aureus (MRSA) from Malaysian hospitals. Journal of Medical Microbiology. 2002;51(12):1113-6.

146. Norazah A, Lim VK, Rohani MY, Kamel AG. In-vitro activity of quinupristin/dalfopristin, levofloxacin and moxifloxacin against fusidic acid and rifampicin-resistant strains of methicillin-resistant Staphylococcus aureus (MRSA) from Malaysian hospitals. The Medical journal of Malaysia. 2005;60(4):411-5.

147. Yildiz T, Çoban AY, Şener AG, Coşkuner SA, Bayramoğlu G, Güdücüoğlu H, et al. Antimicrobial susceptibility and resistance mechanisms of methicillin resistant Staphylococcus aureus isolated from 12 Hospitals in Turkey. Annals of Clinical Microbiology and Antimicrobials. 2014;13(1).

148. Grohs P, Kitzis MD, Gutmann L. In vitro bactericidal activities of linezolid in combination with vancomycin, gentamicin, ciprofloxacin, fusidic acid, and rifampin against Staphylococcus aureus. Antimicrob Agents Chemother. 2003;47(1):418-20.

149. Arkwright PD, Daniel TO, Sanyal D, David TJ, Patel L. Age-related prevalence and antibiotic resistance of pathogenic staphylococci and streptococci in children with infected atopic dermatitis at a single-specialty center. Archives of dermatology. 2002;138(7):939-41.

150. Hoeger PH. Antimicrobial susceptibility of skin-colonizing S-aureus strains in children with atopic dermatitis. Pediatric Allergy and Immunology. 2004;15(5):474-7.

151. Pfaller MA, Castanheira M, Sader HS, Jones RN. Evaluation of the activity of fusidic acid tested against contemporary Gram-positive clinical isolates from the USA and Canada. International Journal of Antimicrobial Agents. 2010;35(3):282-7.

152. Pichon B, Hill RLR, Blackburn R, Ganner M, Harwin L, Cookson B, et al. Molecular epidemiology of Staphylococcus aureus bacteraemia in the UK-2011. Clinical Microbiology and Infection. 2012;18:36.

153. Saginur R, Stdenis M, Ferris W, Aaron SD, Chan F, Lee C, et al. Multiple combination bactericidal testing of staphylococcal biofilms from implant-associated infections. Antimicrob Agents Chemother. 2006;50(1):55-61.

154. Rennie RP. Susceptibility of Staphylococcus aureus to fusidic acid: Canadian data. Journal of cutaneous medicine and surgery. 2006;10(6):277-80.

155. Rijnders M, Nys S, Driessen C, Hoebe CJ, Hopstaken RM, Oudhuis GJ, et al. Staphylococcus aureus carriage among GPs in The Netherlands. The British journal of general practice : the journal of the Royal College of General Practitioners. 2010;60(581):902-6.

156. Flamm RK, Rhomberg PR, Farrell DJ, Jones RN. In vitro spectrum of pexiganan activity; bactericidal action and resistance selection tested against pathogens with elevated MIC values to topical agents. Diagnostic microbiology and infectious disease. 2016;86(1):66-9.

157. Jones RN, Mendes RE, Sader HS, Castanheira M. In vitro antimicrobial findings for fusidic acid tested against contemporary (2008-2009) gram-positive organisms collected in the United States. Clinical infectious diseases : an official publication of the Infectious Diseases Society of America. 2011;52 Suppl 7:S477-86.

158. Rohani MY, Raudzah A, Lau MG, Zaidatul AAR, Salbiah MN, Keah KC, et al. Susceptibility pattern of Staphylococcus aureus isolated in Malaysian hospitals. International Journal of Antimicrobial Agents. 2000;13(3):209-13.

159. Rørtveit S, Rørtveit G. An epidemic of bullous impetigo in an island community in Norway in the year 2002. Tidsskrift for den Norske Laegeforening. 2003;123(18):2557-60.

160. Klein S, Menz MD, Zanger P, Heeg K, Nurjadi D. Increase in the prevalence of Panton–Valentine leukocidin and clonal shift in community-onset methicillin-resistant Staphylococcus aureus causing skin and soft-tissue infections in the Rhine-Neckar Region, Germany, 2012–2016. International Journal of Antimicrobial Agents. 2019;53(3):261-7.

161. Lemaire S, Piérard D, Van Bambeke F, Tulkens P. Intracellular activity of fusidic acid against clinical isolates of Staphylococcus aureus of increasing MIC. Clinical Microbiology and Infection. 2011;17:S182-S3.

162. Koning S, Mohammedamin RSA, Van Der Wouden JC, Van Suijlekom-Smit LWA, Schellevis FG, Thomas S. Impetigo: Incidence and treatment in Dutch general practice in 1987 and 2001 - Results from two national surveys. British Journal of Dermatology. 2006;154(2):239-43.

163. Mehdi SZ, Akber JUD, Nizam M, Dawood K, Buksh AR. Frequency and antimicrobial susceptibility pattern of microorganisms isolated from hospitalized infantile burn cases in a tertiary care hospital. Pakistan Paediatric Journal. 2016;40(3):135-42.

164. Saleem F, Fasih N, Zafar A. Susceptibility pattern of methicillin resistant staphylococcus aureus to vancomycin and other alternate agents: Report from a private sector hospital laboratory. Journal of the Pakistan Medical Association. 2017;67(11):1743-6.

165. Salmanov AG, Shkorbotun VO, Shkorbotun YV. Antimicrobial resistance of staphylococcus aureus causing of surgical site infections in ear, nose and throat surgery. Wiadomosci lekarskie (Warsaw, Poland : 1960). 2019;72(2):154-8.

166. Boswihi SS, Udo EE, Monecke S, Mathew B, Noronha B, Verghese T, et al. Emerging variants of methicillin-resistant Staphylococcus aureus genotypes in Kuwait hospitals. PLoS ONE. 2018;13(4).

167. Perwaiz S, Barakzi Q, Farooqi BJ, Khursheed N, Sabir N. Antimicrobial susceptibility pattern of clinical isolates of methicillin resistant Staphylococcus aureus. JPMA The Journal of the Pakistan Medical Association. 2007;57(1):2-4.

168. Samra Z, Ofer O, Shmuely H. Susceptibility of methicillin-resistant Staphylococcus aureus to vancomycin, teicoplanin, linezolid, pristinamycin and other antibiotics. The Israel Medical Association journal : IMAJ. 2005;7(3):148-50.

169. SǍndulescu O, Grigoraş A, Streinu-Cercel A, Berciu I, Neguţ AC, Streinu-Cercel A. Resistance profile of Staphylococcus aureus strains isolated from patients treated in a tertiary care hospital in Romania. BMC Infectious Diseases. 2014;14(7).

170. Park SH, Kim JK, Park K. In Vitro Antimicrobial Activities of Fusidic Acid and Retapamulin against Mupirocin- and Methicillin-Resistant Staphylococcus aureus. Annals of Dermatology. 2015;27(5):551-6.

171. Sasirekha B, Usha MS, Amruta AJ, Ankit S, Brinda N, Divya R. Evaluation and comparison of different phenotypic tests to detect Methicillin resistant Staphylococcus aureus and their biofilm production. International Journal of PharmTech Research. 2012;4(2):532-41.

172. Scerri J, Monecke S, Borg MA. Prevalence and characteristics of community carriage of methicillin-resistant staphylococcus aureus in malta. Journal of Epidemiology and Global Health. 2013;3(3):165-73.

173. Senok AC, Somily A, Raji M, Garaween G, Kabil M, Shibl A, et al. Genotyping of staphylococcus aureus associated with nasal colonization among healthcare workers using DNA microarray. Journal of Infection in Developing Countries. 2018;12(5):321-5.

174. Nishijima S, Kurokawa I. Antimicrobial resistance of Staphylococcus aureus isolated from skin infections. Int J Antimicrob Agents. 2002;19(3):241-3.

175. Seydi M, Sow AI, Soumaré M, Diallo HM, Hatim B, Tine R, et al. Staphylococcus aureus bacteremia in the Dakar Fann university hospital. Medecine et Maladies Infectieuses. 2004;34(5):210-5.

176. Sfeir M, Obeid Y, Eid C, Saliby M, Farra A, Farhat H, et al. Prevalence of Staphylococcus aureus methicillin-sensitive and methicillin-resistant nasal and pharyngeal colonization in outpatients in Lebanon. American Journal of Infection Control. 2014;42(2):160-3.

177. Tunger O, Arisoy A, Kurutepe S, Akcali S, Ozbakkaloglu B. In vitro susceptibility of Staphylococcus aureus and coagulase-negative Staphylococcus strains to fusidic acid. Int J Antimicrob Agents. 2001;18(5):445-7.

178. Souli M, Karaiskos I, Galani L, Maraki S, Perivolioti E, Argyropoulou A, et al. Nationwide surveillance of resistance rates of Staphylococcus aureus clinical isolates from Greek hospitals, 2012-2013. Infectious Diseases. 2016;48(4):287-92.

179. Stevens CL, Ralph A, McLeod JE, McDonald MI. Community-acquired methicillin-resistant Staphylococcus aureus in Central Australia. Communicable diseases intelligence quarterly report. 2006;30(4):462-6.

180. Strandén A, Frei R, Widmer AF. Lack of emergence of PVL-positive MRSA strains in a university hospital. Clinical Microbiology and Infection. 2012;18:339.

181. Sule O, Brown N, Brown DFJ, Burrows N. Fusidic acid cream for impetigo - Judicious use is advisable. British Medical Journal. 2002;324(7350):1394-5.

182. Rortveit S, Skutlaberg DH, Langeland N, Rortveit G. The decline of the impetigo epidemic caused by the epidemic European fusidic acid-resistant impetigo clone: an 11.5-year population-based incidence study from a community in Western Norway. Scand J Infect Dis. 2014;46(12):832-7.

183. Rørtveit S, Skutlaberg DH, Langeland N, Rortveit G. Impetigo in a population over 8.5 years: Incidence, fusidic acid resistance and molecular characteristics. Journal of Antimicrobial Chemotherapy. 2011;66(6):1360-4.

184. Swathi G, Chandan KS, Murgesh SB. Clinical and bacteriological study of pyoderma with reference to antibiotic susceptibility profile. Journal of Pakistan Association of Dermatologists. 2019;29(1):83-8.

185. Titov L, Ermakova T, Gorbunov V, Lebedev F, Kazakov I, Glazkova S. Methicillin-resistant staphylococci in the Republic of Belarus: Results of the National Surveillance System (2008-2010). Clinical Microbiology and Infection. 2011;17:S198-S9.

186. Treesirichod A, Hantagool S, Prommalikit O. Nasal carriage and antimicrobial susceptibility of Staphylococcus aureus among medical students at the HRH Princess Maha Chakri Sirindhorn Medical Center, Thailand: a cross sectional study. J Infect Public Health. 2013;6(3):196-201.

187. Tuncer Ertem G, Öztürk B, Ataman HatIpoǧlu C, Ipekkan K, Erdem F, Adiloǧlu AK, et al. In vitro susceptibilities of staphylococcus and enterococcus isolates to linezolid, daptomycin, teicoplanin and fusidic acid. Turkiye Klinikleri Journal of Medical Sciences. 2013;33(6):1381-7.

188. Tveten Y, Jenkins A, Allum AG, Kristiansen BE, Norwegian MSG. Heterogeneity of methicillin-resistant Staphylococcus aureus isolated in Norway. Clinical Microbiology and Infection. 2003;9(8):886-92.

189. Udo E, Boswihi S, AlFouzan W, Mathew B, Noronha B, Verghese T, et al. Stability of the dominant Methicillin-resistant Staphylococcus aureus clones in Kuwait hospitals in 2016–2017. Journal of Infection and Public Health. 2019;12(1):141.

190. Udo EE, Al-Lawati BAH, Al-Muharmi Z, Thukral SS. Genotyping of methicillin-resistant Staphylococcus aureus in the Sultan Qaboos University Hospital, Oman reveals the dominance of Panton-Valentine leucocidin-negative ST6-IV/t304 clone. New Microbes and New Infections. 2014;2(4):100-5.

191. Udo EE, Al-Sweih N, Dhar R, Dimitrov TS, Mokaddas EM, Johny M, et al. Surveillance of antibacterial resistance in Staphylococcus aureus isolated in Kuwaiti hospitals. Medical principles and practice : international journal of the Kuwait University, Health Science Centre. 2008;17(1):71-5.

192. Uluǧ M, Ayaz C, Çelen MK. The importance of fusidic acid in the treatment of chronic osteomyelitis. Anatolian Journal of Clinical Investigation. 2009;3(4):222-6.

193. Vallières E, Rendall JC, Moore JE, McCaughan J, Tunney MM, Elborn JS, et al. MRSA eradication in CF patients with lower respiratory tract infection. Journal of Cystic Fibrosis. 2015;14:S83.

194. Gostev V, Kruglov A, Kalinogorskaya O, Dmitrenko O, Khokhlova O, Yamamoto T, et al. Molecular epidemiology and antibiotic resistance of methicillin-resistant Staphylococcus aureus circulating in the Russian Federation. Infection, genetics and evolution : journal of molecular epidemiology and evolutionary genetics in infectious diseases. 2017;53:189-94.

195. Vourli S, Perimeni D, Makri A, Polemis M, Voyiatzi A, Vatopoulos A. Community acquired MRSA infections in a paediatric population in Greece. Euro surveillance : bulletin Europeen sur les maladies transmissibles = European communicable disease bulletin. 2005;10(5):78-9.

196. Alfouzan W, Udo EE, Modhaffer A, Alosaimi A. Molecular Characterization of Methicillin- Resistant Staphylococcus aureus in a Tertiary Care hospital in Kuwait. Scientific reports. 2019;9(1):18527.

197. Wasserman E, Orth H, Senekal M, Harvey K. High prevalence of mupirocin resistance associated with resistance to other antimicrobial agents in Staphylococcus aureus isolated from patients in private health care, Western Cape. Southern African Journal of Epidemiology and Infection. 2014;29(4):126-32.

198. Wang WY, Chiueh TS, Lee YT, Tsao SM. Correlation of molecular types with antimicrobial susceptibility profiles among 670 meca-positive MRSA isolates from sterile sites (tist study, 2006-2010). Journal of Microbiology, Immunology and Infection. 2015;48(2):S39.

199. Wiśniewska K, Piechowicz L, Galiński J. Predominance of multidrug resistant strains with reduced susceptibility to fusidic acid among methicillin-resistant Staphylococcus aureus strains (MRSA) isolated in the Gdánsk region. Polski merkuriusz lekarski : organ Polskiego Towarzystwa Lekarskiego. 2000;9(53):746-50.

200. Wiśniewska K, Dajnowska-Stanczewa A, Galiński J, Garbacz K. Methicillin-resistant Staphylococcus aureus (MRSA) with high resistance to mupirocin in hospitals of the Gdańsk region. Medycyna doświadczalna i mikrobiologia. 2002;54(4):285-92.

201. Liu X, Deng S, Huang J, Huang Y, Zhang Y, Yan Q, et al. Dissemination of macrolides, fusidic acid and mupirocin resistance among Staphylococcus aureus clinical isolates. Oncotarget. 2017;8(35):58086-97.

202. Chen X, Yang HH, Huangfu YC, Wang WK, Liu Y, Ni YX, et al. Molecular epidemiologic analysis of Staphylococcus aureus isolated from four burn centers. Burns. 2012;38(5):738-42.

203. Yilmaz ES, Aslantas O. Antimicrobial resistance and underlying mechanisms in Staphylococcus aureus isolates. Asian Pacific Journal of Tropical Medicine. 2017;10(11):1059-64.

204. Liu Y, Xu Z, Yang Z, Sun J, Ma L. Characterization of community-associated Staphylococcus aureus from skin and soft-tissue infections: a multicenter study in China. Emerging microbes & infections. 2016;5(12):e127.

205. Baek YS, Jeon J, Ahn JW, Song HJ. Antimicrobial resistance of Staphylococcus aureus isolated from skin infections and its implications in various clinical conditions in Korea. Int J Dermatol. 2016;55(4):e191-7.

206. Huang YT, Liao CH, Chen SY, Yang CJ, Hsu HS, Teng LJ, et al. Characterization of rifampin-resistant staphylococcus aureus nasal carriage in patients receiving rifampin-containing regimens for tuberculosis. Infection and Drug Resistance. 2018;11:1175-82.

207. Rashid Z, Sattar A, Qureshi MIM, Farzana K, Rashid F, Murtaza G. Nasal carriage of staphylococci in medical personnel, sanitary workers and non-medical personnel. Latin American Journal of Pharmacy. 2012;31(10):1496-500.

208. Zinn CS, Westh H, Rosdahl VT. An international multicenter study of antimicrobial resistance and typing of hospital Staphylococcus aureus isolates from 21 laboratories in 19 countries or states. Microb Drug Resist. 2004;10(2):160-8.

209. Willey B, Gnanasuntharam P, Rostas A, Porter V, Kreiswirth N, Louie L, et al. Molecular diversity of community-acquired Methicillin-Resistant Staphylococcus Aureus (MRSA) in Toronto. International Journal of Antimicrobial Agents. 2009;34:S110-S1.

210. Bhattacharya S, Pal K, Jain S, Chatterjee SS, Konar J. Surgical Site Infection by Methicillin Resistant Staphylococcus aureus- on Decline? Journal of Clinical and Diagnostic Research. 2016;10(9):DC32-DC6.

211. Chang CH, Lin TC, Chang CH, Hong SJ, Tsai YC. Methicillin-resistant Staphylococcus aureus in skin and soft tissue infections and minocyclin treatment experience in the dermatological setting of eastern Taiwan. Dermatologica Sinica. 2011;29(3):86-90.

212. Hwang JH, Tsai HY, Liu TC. Community-acquired methicillin-resistant Staphylococcus aureus infections in diskharging ears. Acta oto-laryngologica. 2002;122(8):827-30.

213. Kareiviene V, Pavilonis A, Sinkute G, Liegiute S, Gailiene G. Staphylococcus aureus resistance to antibiotics and spread of phage types. Medicina (Kaunas, Lithuania). 2006;42(4):332-9.

214. Sklyar TV, Lavrentieva KV, Gavrilyuk VG, Kurahina NV, Vereshchaha MO, Lykholat OA. Monitoring of multiresistant community-associated MRSA strains from patients with pathological processes of different localization. Regulatory Mechanisms in Biosystems. 2018;9(2):281-6.

215. Ibrahim N, Siti-Fairuz MH, Nurul-Iwani MAA. A survey on community acquired-meticillin resistant Staphylococcus aureus burden among university students. International Journal of Infectious Diseases. 2012;16:e223.
